# Designing metal chelates of halogenated sulfonamide Schiff bases as potent nonplatinum anticancer drugs using spectroscopic, molecular docking and biological studies

**DOI:** 10.1038/s41598-022-24512-y

**Published:** 2022-11-23

**Authors:** Rehab M. I. Elsamra, Mamdouh S. Masoud, Ahmed M. Ramadan

**Affiliations:** Chemistry Department, Faculty of Science, Alexandria University, P.O. Box 426, Alexandria, 21321 Egypt

**Keywords:** Cancer, Computational biology and bioinformatics, Chemistry

## Abstract

In this contribution, five Ni(II) complexes have been synthesized from sulfonamide-based Schiff bases (SB^1^–SB^5^) that comprise bromo or iodo substituents in the salicylidene moiety. The chemical structures of these compounds were extensively elucidated by different analytical and physicochemical studies. All ligands act as bidentate chelators with ON binding mode yielding octahedral, square planar, or tetrahedral geometries. The phenolic OH at δ 12.80 ppm in the free Schiff base SB^2^ vanishes in the ^1^H NMRspectrum of diamagnetic complex [Ni(SB^2^–H)_2_] favoring the OH deprotonation prior to the chelation with Ni(II) ion. The appearance of twin molecular ion peaks ([M − 1]^+^ and [M + 1]^+^) is due to the presence of bromine isotopes (^79^Br and ^81^Br) in the mass spectra of most cases. Also, the thermal decomposition stages of all complexes confirmed their high thermal stability and ended with the formation of NiO residue of mass 6.42% to 14.18%. Besides, antimicrobial activity and cytotoxicity of the ligands and some selected complexes were evaluated. Among the ligands, SB^4^ showed superior antimicrobial efficacy with MIC values of 0.46, 7.54, and 0.95 µM against *B. subtilis*, *E. coli*, and *A. fumigatus* strains, respectively. The consortium of different substituents as two bromine atoms either at positions 3 and/or 5 on the phenyl ring and a thiazole ring is one of the reasons behind the recorded optimal activity. Moreover, there is a good correlation between the cytotoxicity screening (IC_50_) and molecular docking simulation outcomes that predicted a strong binding of SB^2^ (16.0 μM), SB^4^ (18.8 μM), and SB^5^ (6.32 μM) to the breast cancer protein (*3s7s*). Additionally, [Ni(SB^4^–H)_2_] (4.33 µM) has nearly fourfold potency in comparison with cisplatin (19.0 μM) against breast carcinoma cells (MCF-7) and is highly recommended as a promising, potent, as well as low-cost non-platinum antiproliferative agent after further drug authorization processes.

## Introduction

Conjugated heterocyclic systems containing nitrogen, oxygen, and sulfur atoms in their structures have long been known for their diverse biological actions^1–3^. The activity of these heterocyclic compounds against wide varieties of pathogens increases especially when introduced in the form of metal complexes^4,5^. Transition metal complexes of Schiff bases derived from sulfa drugs are among systems that have been reported extensively for their pronounced medicinal performance^5–8^. Most of the sulfa drugs exhibit a bacteriostatic effect by preventing cell division through the inhibition of the dihydropteroate synthase enzyme that is essential in the proteins and nucleic acids formation in bacterial cells^9,10^. Crucially, coupling of Schiff bases-sulfonamide moieties with halogen substituents could enhance the therapeutic efficiency including antimicrobial, antitumor, and antiviral activities^11–14^. Halogens, such as bromine and iodine have a prominent ability to interact with electron donor atoms due to their electron-withdrawing property that generates σ-hole, positive electrostatic potential, along the halogen bond ^15^. They can effectively form stable donor–acceptor bonds with the surrounding molecules such as the electron-rich sites of the adjacent amino acids. This enhances the binding affinity to certain proteins^16^.

Clearly, the mutual existence of different units of hetero atoms, halogens, and metal ions in one combined structure is particularly important because of the role played by each entity. Such combined structure creates unequal electron density distribution with different electrophilic and nucleophilic regions on the surface of the molecules which increases the chance of chelation with proteins and provides a wide range of chemical and biological practices. Although there are massive investigations on antipathogenic agents with different functional groups and structures to find more effective and less toxic drugs, the rapid evolution of research is still challenging the need to overcome the quick development of drug-resistant microbes. Besides, Schiff bases have been recognized as catalysts^17^, corrosion inhibitors^18^, and efficient polymer stabilizers^19^. The diversity of the bioinorganic applications of Schiff bases is stemmed from the fact that this class of compounds possesses a high complexing ability through the azomethine group and neighboring donor atoms in a polydentate fashion^20–23^. In consideration of the mentioned observations, the current research is aiming to examine the microbial and tumor inhibition activities of newly synthesized nickel (II) complexes derived from mono- and di-halogenated Schiff bases bearing the bioactive benzenesulfonamide group. In addition, the structural variations of the investigated ligands and complexes such as different pendant substituents or geometric chelation types are correlated theoretically and experimentally as important parameters accountable for their chemical and biological behaviors.

## Experimental

### Chemicals, instruments, and computations

Nickel (II) nitrate hexahydrate salt and 5-bromosalicylaldehyde were obtained from Sigma-Aldrich with high purity (≥ 98%). 3,5-Dihalosubstituted salicylaldehyde (X_2_C_6_H_2_(*o*-OH)CHO), where X = Br or I, were prepared following the known methods in the literature^24,25^. Infrared spectra of the synthesized ligands and their complexes were recorded by FT-IR tensor 37 spectrophotometer in the range 400–4000 cm^−1^. Microanalysis (CHNS) was performed at the microanalytical laboratory, Cairo University. The metal content in each sample was doubly determined by atomic absorption spectrophotometer and by complexometric back titration using standard EDTA solution and Eriochrome black T indicator^26^. Also, the absorption in the UV–Vis region was conducted by the SHIMADZU UV-1800 scanning spectrophotometer in the wavelength range 200–800 nm using dimethylformamide as a solvent. The mass-to-charge ratio of the molecular ions was measured by SHIMADZU mass spectrometer (QP2010 Plus) at an electron energy of 70 eV at Cairo University and by Thermo Scientific GCMS model ISQ et al.-Azhar University, Cairo, Egypt. Thermal decomposition of the complexes was investigated by the thermogravimetric (TGA) as well as the differential thermogravimetric (DTA) techniques using LINSEIS STA PT1000 analyzer under N_2_ atmosphere as inert gas and a temperature rate of 10 °C/min. The ^1^H NMR and ^13^C NMR spectra were studied for the ligands and the diamagnetic complex in a deuterated dimethyl sulfoxide solvent on a 500 MHz spectrometer.

Geometry optimization and structural energy calculations of some selected compounds were computed by density functional theory (DFT) using GAUSSIAN09 software, Version 9.5, and GAUSSVIEW 6.0.16^27,28^. Hybrid exchange–correlation function (B3LYP) with the basis set Lanl2dz was chosen for the investigated systems. This level of theory (DFT/B3LYP) has been approved as an effective approach to calculate the electronic properties of organic systems containing electronegative atoms, analogous to the present work^29,30^. True energy minima of the optimized structures were proved by the absence of imaginary vibrational frequencies in the GAUSSIAN output files. A molecular docking study was conducted by the Molecular Operating Environmental module (MOE 2015.10). The 3D structure of the selected protein *3s7s* was adopted from the protein data bank. As docking initial steps, the protein structure was set up by removing water molecules and adding hydrogen atoms. Also, a site finder was used for the ligand-binding site prediction. Evaluation of the best binding pose between the investigated ligands and the receptor protein was based on the H-bond length and the scoring energy of the simulated docked complex.

### Antimicrobial and cytotoxicity assessment

The in vitro biological activity of the synthesized halogenated Schiff bases and some of their complexes was investigated employing Kirby–Bauer agar diffusion assay against different pathogenic species including Gram-negative bacteria (*P. vulgaris* & *E. coli*), Gram-positive bacteria (*S. aureus* & *B. subtilis*), and fungi (*A. fumigatus* & *C. albicans*) as depicted in the literature^31^. All inhibition zone diameter values in mm were assessed in triplicates for the tested samples. Also, MIC (minimal inhibitory concentration in µg/ml) was determined by the serial dilution method as reported^32,33^. The activity of the tested compounds was compared to ketoconazole, gentamycin, and ampicillin as reference controls for antifungal and antibacterial potencies.

Besides, the cytotoxicity of some selected synthesized ligands and complexes against human breast carcinoma cell line (MCF-7) and normal human oral epithelial cell (OEC) was evaluated by colorimetric viability assay as reported^34^. All human cancer cell lines were obtained from the VACSERA Tissue Culture Unit, Egypt and the OEC cells (PCS-200-014) were from American Culture Type Collection. Cisplatin was used as a standard reference under the same assay specifications. The biological measurements were carried out et al.-Azhar University, Cairo, Egypt.

### Synthesis of Schiff bases (SB^1^–SB^5^)

**Figure 1:**
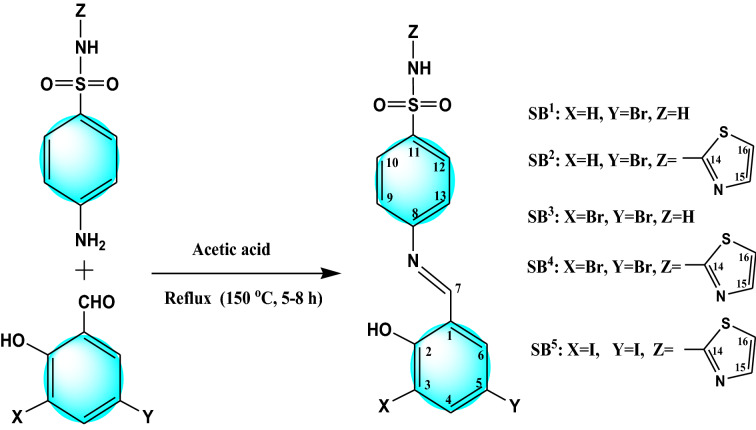
Systematic path for the synthesis of sulfonamide-Schiff base ligands (SB^1^–SB^5^).

Mono- or dihalosubstituted salicylaldehyde (10 mmol) was dissolved in ethanol and then added to equimolar of a primary amine solution, sulfanilamide (10 mmol, 1.7220 g) or sulfathiazole (10 mmol, 2.5552 g). A few drops of acetic acid were added as a catalyst. The reaction mixture was refluxed with continuous stirring for 5–8 h in a water bath. The colored Schiff bases were filtered off, washed by ethanol and ether then dried in a calcium chloride desiccator. Recrystallization of the resulted solids was performed in hot ethanol–water solvents (1:1) for complete purification. The synthesized sulfonamide-Schiff bases are presented in Fig. 1. The IUPAC names of the synthesized ligands are 4-((5-Bromo-2-hydroxybenzylidene)amino)-benzenesulfonamide (SB^1^), 4-((5-Bromo-2-hydroxybenzylidene)amino)-*N*-(1,3-thiazol-2-yl)benzenesulfonamide (SB^2^), 4-((3,5-Dibromo-2-hydroxybenzylidene)amino)-benzenesulfonamide (SB^3^), 4-((3,5-Dibromo-2-hydroxybenzylidene)amino)-N-(1,3-thiazol-2-yl)benzenesulfonamide (SB^4^), and 4-((3,5-Diiodo-2-hydroxybenzylidene)amino)-N-(1,3-thiazol-2-yl)benzenesulfonamide (SB^5^).

### Synthesis of nickel (II) complexes

Ni (II) complexes were prepared by mixing an aqueous solution of Ni(NO_3_)_2_.6H_2_O (1.4533 g, 5 mmol) with hot ethanolic solution of Schiff bases (SB^1^–SB^5^) (10 mmol) under stirring and reflux at 70 °C for 2 h. The solution was turned to a slightly basic medium (pH ≈ 8) by adding a few drops of ammonia (1:1). The yielded complexes were filtered, washed with a small amount of ethanol and ether, then kept in desiccators for drying. Elemental analysis and some physical properties of the synthesized ligands and their complexes are specified in Table 1.

**Table 1:** Elemental analysis and physical properties of the synthesized nickel (II) complexes. All complexes have m.p. > 300 °C.

Compound	Formula Color, m.p. °C	% Calculated/(Found)					*μ*_eff_ B.M	Geometry
		M	C	H	N	S		
SB^1^	C_13_H_11_BrN_2_O_3_S Orange, 184–186	–	43.96 (44.12)	3.12 (3.32)	7.89 (7.96)	9.03 (8.82)	–	–
[Ni(SB^1^–H)_2_(SB^1^)]·2H_2_O	C_39_H_35_Br_3_N_6_O_11_S_3_Ni Yellow	5.07 (4.89)	40.44 (40.62)	3.05 (3.15)	7.26 (7.43)	8.30 (8.22)	3.13	Oh
SB^2^	C_16_H_12_BrN_3_O_3_S_2_ Yellow, 162–164	–	43.84 (43.97)	2.76 (2.89)	9.59 (9.73)	14.63 (14.32)	–	–
[Ni( SB^2^–H)_2_]·3H_2_O	C_32_H_28_Br_2_N_6_O_9_S_4_Ni Greenish yellow	5.94 (5.84)	38.93 (39.05)	2.86 (3.01)	8.51 (8.82)	12.99 (12.81)	dia	SP
SB^3^	C_13_H_10_Br_2_N_2_O_3_S Red, 242–244	–	35.97 (36.12)	2.32 (2.35)	6.45 (6.43)	7.39 (7.22)	–	–
[Ni(SB^3^–H)(OH)(H_2_O)]	C_13_H_12_Br_2_N_2_O_5_SNi Yellowish green	11.14 (11.02)	29.64 (29.71)	2.30 (2.62)	5.32 (5.59)	6.09 (5.98)	0.50	SP
SB^4^	C_16_H_11_Br_2_N_3_O_3_S_2_ Buff, 141–142	–	37.16 (37.42)	2.14 (2.35)	8.12 (8.41)	12.40 (12.22)	–	–
[Ni( SB^4^–H)_2_]·4H_2_O	C_32_H_28_Br_4_N_6_O_10_S_4_Ni Bright yellow	5.05 (4.88)	33.04 (33.29)	2.43 (2.56)	7.23 (7.42)	11.03 (10.92)	2.91	Td
SB^5^	C_16_H_11_I_2_N_3_O_3_S_2_ Brown, 243–245	–	31.44 (31.62)	1.81 (2.15)	6.87 (7.10)	10.49 (10.22)	–	–
[Ni(SB^5^–H)(OH)(H_2_O)]	C_16_H_13_I_2_N_3_O_5_S_2_Ni Yellowish green	8.34 (8.55)	27.30 (27.47)	1.86 (2.06)	5.97 (6.22)	9.11 (9.04)	2.15	SP ↔ Td

## Results and discussion

### FT-IR, NMR, and MS spectroscopy

**Figure 2:**
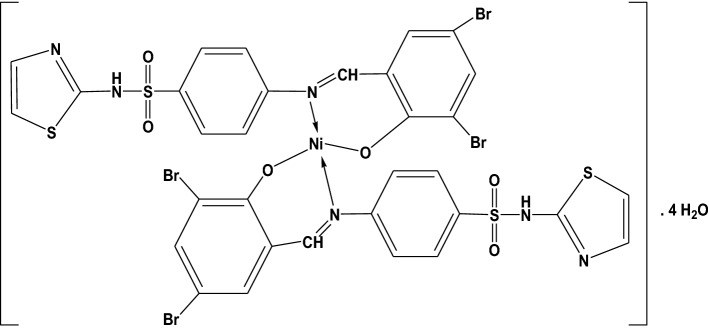
Structure of [Ni(SB^4^–H)_2_]·4H_2_O complex.

Some fundamental FT-IR bands of the free ligands (SB^1^–SB^5^) and the synthesized Ni(II) complexes are compared in their position and shape as presented in Table S1 and illustrated in Figs. 1 and S1–S6. It is discernible from the spectral data that the assigned bands for the νCH=N and νC–O in the free ligands at 1559–1584 cm^−1^ and 1232–1279 cm^−1^, respectively, both suffer a lowering in intensity and wavenumbers supporting the bidentate coordination mode towards the Ni(II) through the phenolic oxygen and the nitrogen atom of the azomethine group (Fig. 2)^13^.

**Figure 3:**
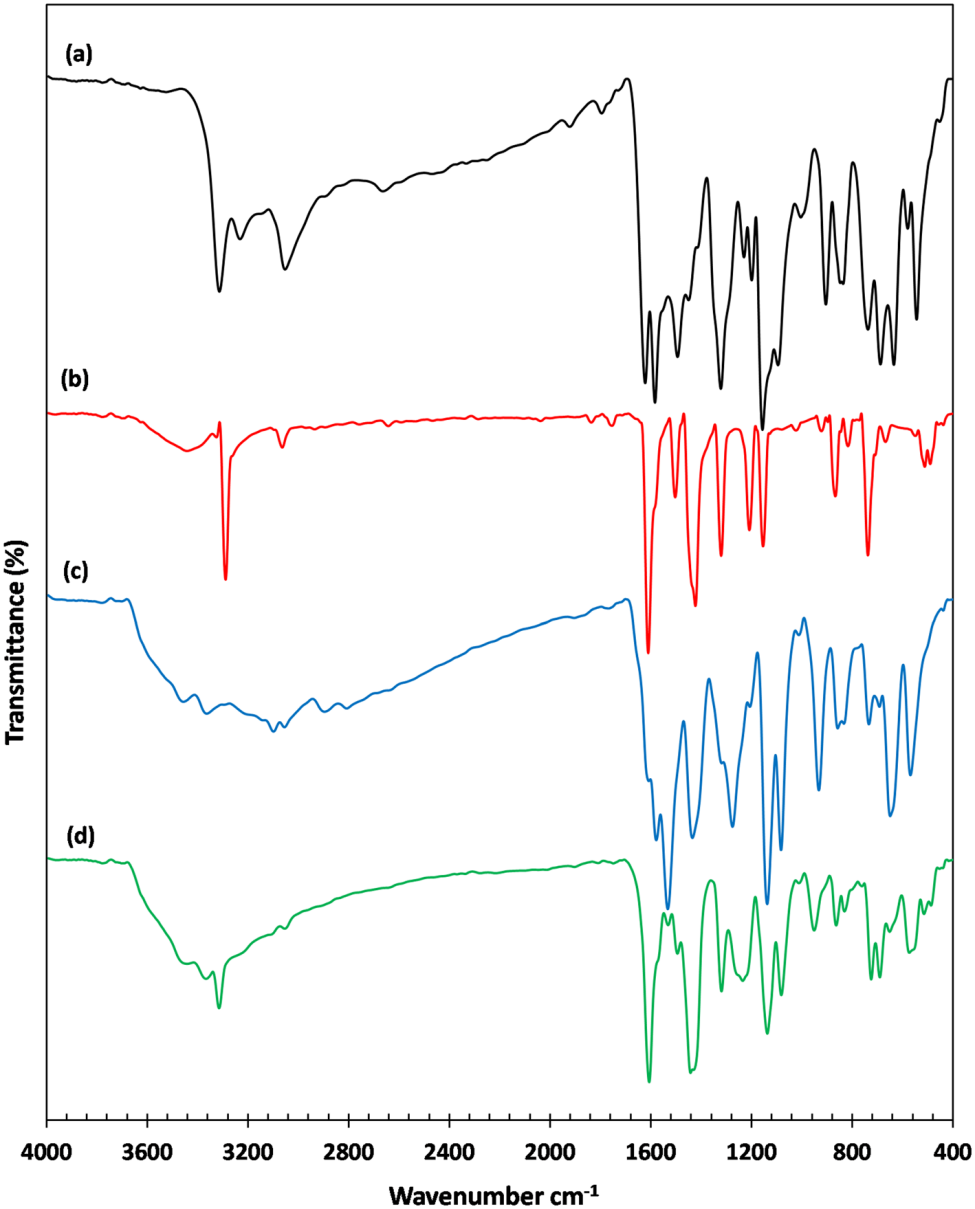
FT-IR of (**a**) SB^3^, (**b**) [Ni(SB^3^–H)(OH)(H_2_O)], (**c**) SB^5^, (**d**) [Ni(SB^5^–H)(OH)(H_2_O)].

In the case of SB^1^ and SB^3^ ligands derived from sulfonamide moiety, typical adjacent bands of asymmetric and symmetric νNH_2_ were observed at 3314–3359 cm^−1^ and 3232–3270 cm^−1^, successively. Also, two stretching bands of the SO_2_ group of these ligands were detected in 1318–1336 cm^−1^ and 1138–1157 cm^−1^ for the asymmetric and symmetric vibrational modes, respectively. No change in the band’s position of NH_2_ or SO_2_ was discerned upon complexation which excludes these groups from being involved in the binding mode with the central metal ion. As for the ligands derived from sulfathiazole (SB^2^, SB^4^, and SB^5^), the inference of the exact change in position or the disappearance of the νO–H band of the phenolic group upon complexation was not straightforward due to overlapping with the νN–H band in the spectral region (3053–3457 cm^−1^) as depicted in Table S1. However, some remarkable changes in this spectral region were spotted. For example, the broad feature in this region could be attributed to intramolecular hydrogen-bonding (O–H–N) with the nearby azomethine group in ligands or of (O–H–O) type with water molecules inside or outside the coordination sphere^35^. In addition, the new broad bands assigned for νO–H at ~ 3440 cm^−1^ in the IR spectra of [Ni(SB^3^–H)(OH)(H_2_O)] and [Ni(SB^5^–H)(OH)(H_2_O)] complexes, Fig. 3, could endorse the existence of covalently bonded OH group in their inner spheres from the alkaline medium as previously reported^36^. Furthermore, the isolated complexes showed extra vibrational bands in the ranges 506–566 cm^−1^ and 458–506 cm^−1^, where these developed bands were assigned to the Ni–O and Ni–N stretching modes, respectively, indicating the proposed chelation mode^37^.

**Figure 4:**
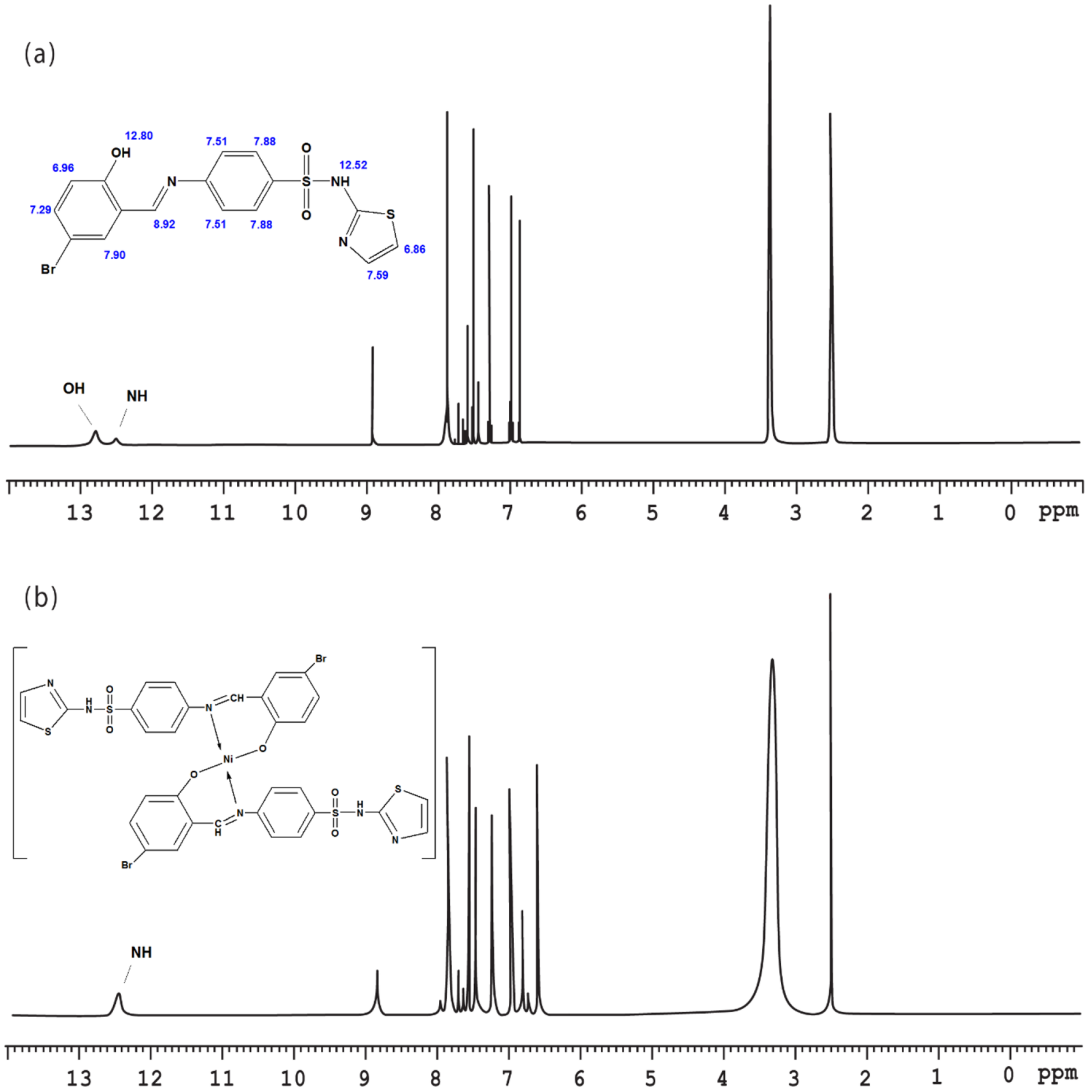
^1^H NMR spectra of (**a**) SB^2^, (**b**) [Ni(SB^2^–H)_2_] complex in DMSO-*d*_6_.

^1^H NMR and ^13^C NMR are additional techniques that support plentifully the structure elucidation of the synthesized compounds. The NMR data are collected in Figs. 4 and S7–S15 in the supplementary information with detailed assignments on each designated structure. Regarding the fundamental ^1^H NMR peaks, the phenolic OH at C(2) in the free Schiff base ligands (SB^1^–SB^5^) were located within the range of δ 12.53–14.26 ppm. This peak at δ 12.80 ppm in SB^2^ vanishes in the diamagnetic complex [Ni(SB^2^–H)_2_] (Fig. 4) favoring the OH deprotonation prior to the chelation with Ni(II) ion. The presence of halogen atoms (Br or Cl) at positions C(3) and/or C(5) (Fig. 1) with an electron-withdrawing feature may facilitate the proton elimination at C(2). However, the NH peak of the sulfathiazole moiety in SB^2^, SB^4^, and SB^5^ which appeared at δ 12.52–12.84 ppm exhibits no change upon complexation. Also, the two protons of the sulfanilamide NH_2_ group of SB^1^ and SB^3^ were observed in the aromatic region at δ 7.42 and 7.38 ppm, respectively. Moreover, Schiff base formation was further supported by the spotted azomethine proton (C7(H) = N) as a singlet peak in the range δ 8.86–9.01 ppm. Pertain to ^13^C NMR, the compound SB^2^, C_16_H_12_N_3_O_3_S_2_Br, showed 14 different carbon peaks of which two possess integration corresponding to 2 identical carbons at 122.1 and 127.2 ppm for [C(9), C(13] and [C(10), C(12)], respectively. Further, the position of halogen-bearing carbons [C(3) and C(5)] is clearly downfield shifted ongoing from SB^4^ (Fig. S13) to SB^5^ (Fig. S15) owing to the higher deshielding effect by Br atoms [C(3) 111.2; C(5) 112.4 ppm] relative to that induced by I atoms [C(3) 88.9; C(5) 82.1 ppm].

**Figure 5:**
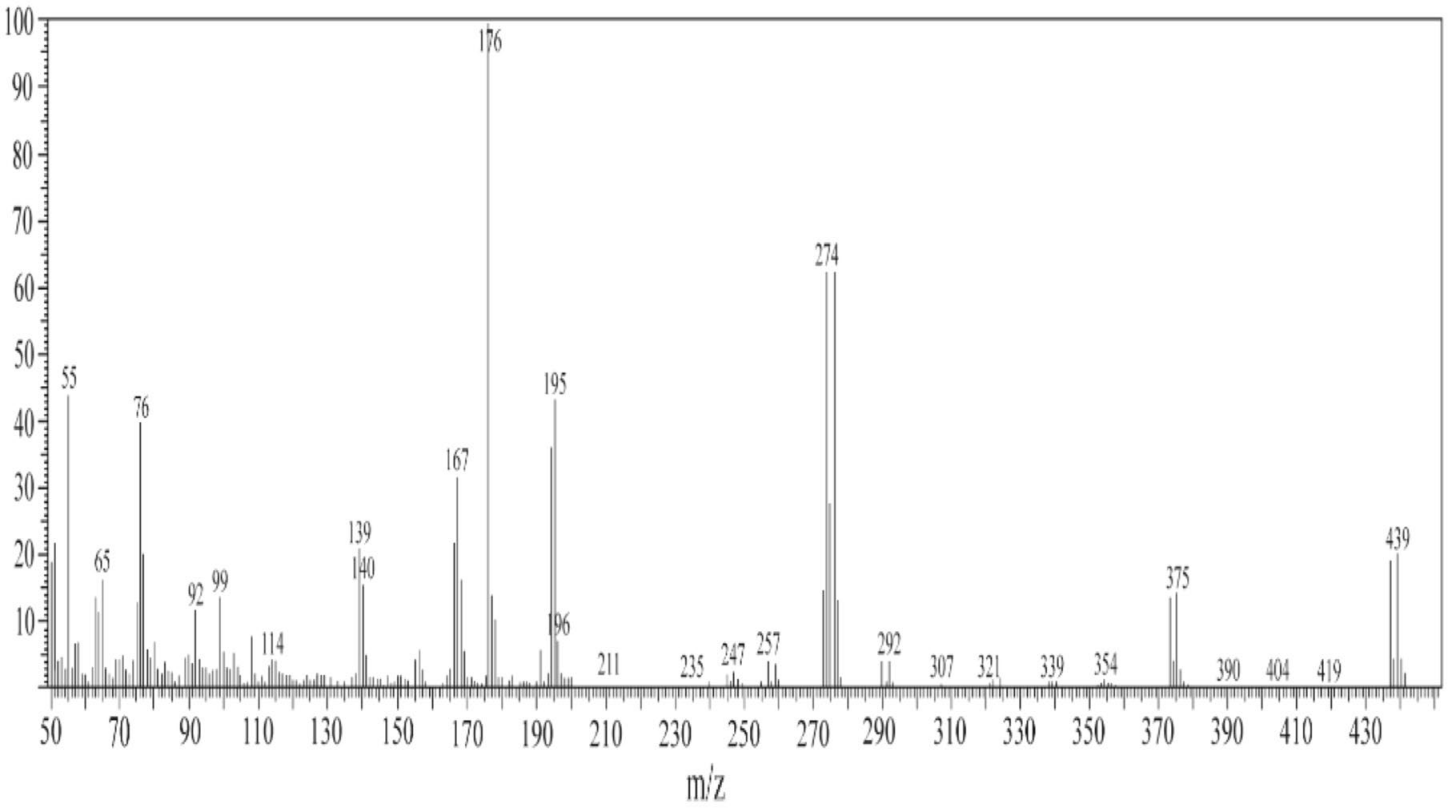
Mass spectrum of SB^2^.

**Figure 6:**
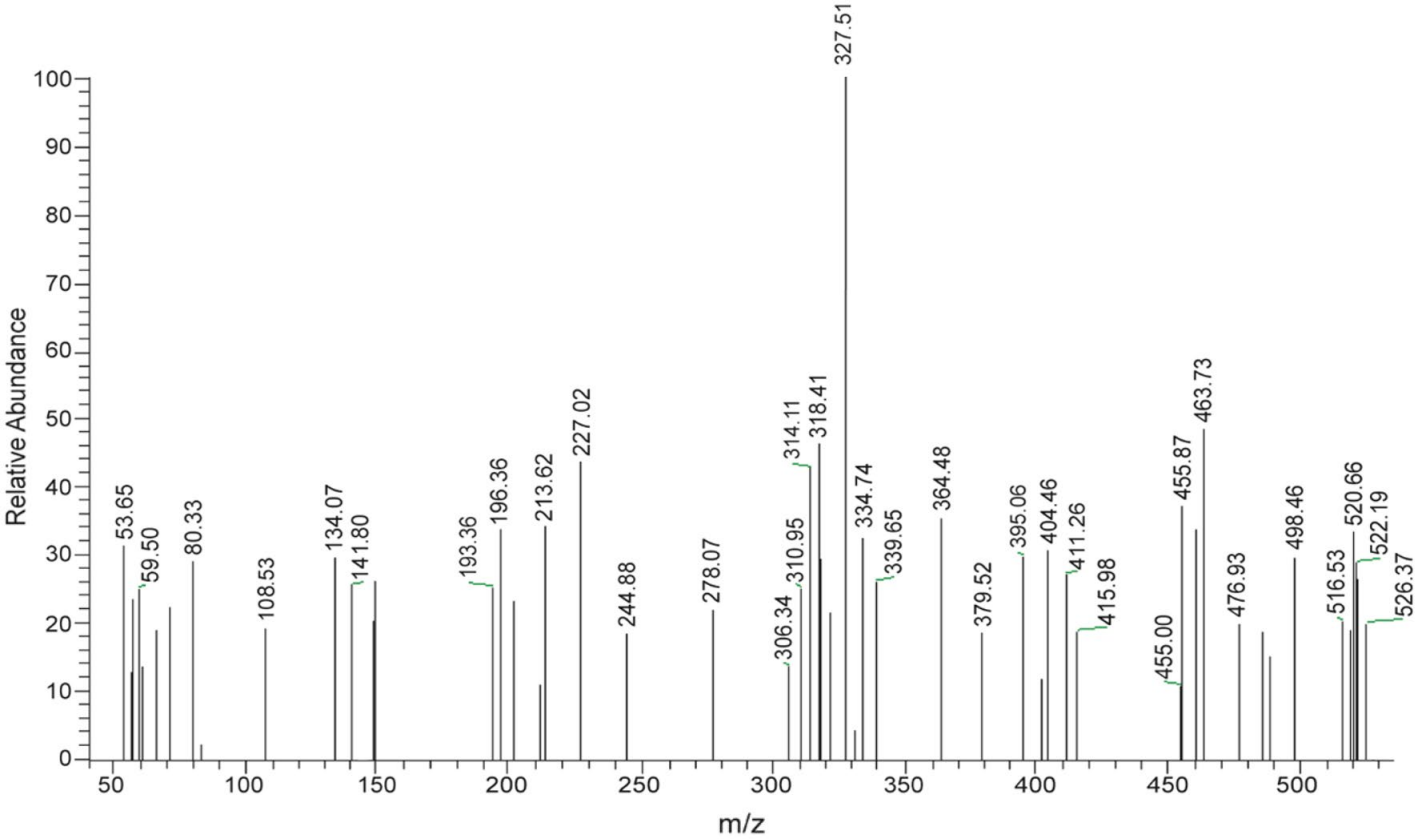
MS of [Ni(SB^3^–H)(OH)(H_2_O)] complex.

**Figure 7:**
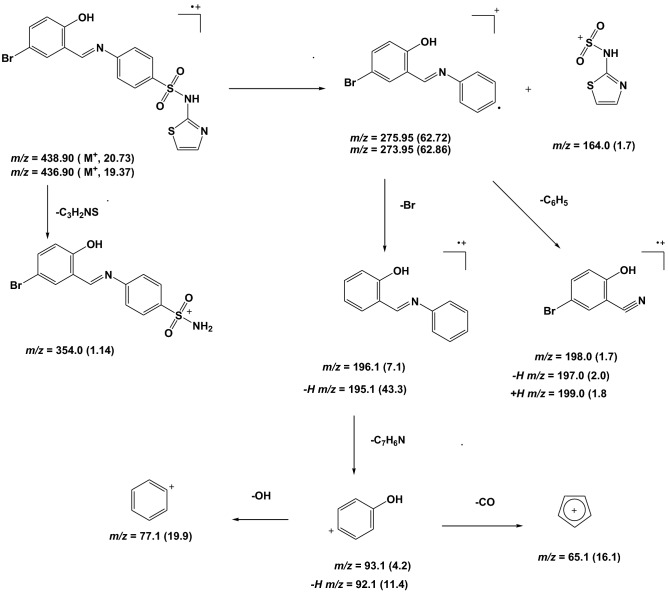
Proposed mass fragmentation SB^2^ ligand.

**Figure 8:**
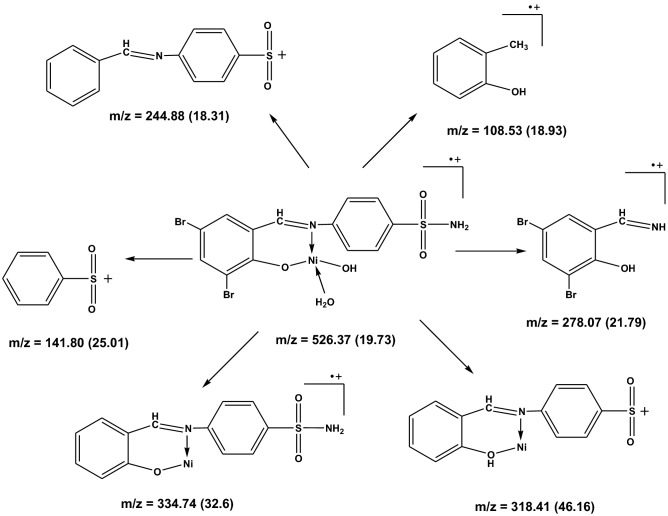
Proposed mass fragmentation of [Ni(SB^3^–H)(OH)(H_2_O)] complex.

The structures of some selected ligands and complexes were further elucidated by the mass spectrometric technique. The mass-to-charge ratio (*m/z*) is displayed in Figs. 5, 6 and S16–S18, where the recorded molecular ion peaks are in accordance with the determined formula mass from the microanalytical technique. The *m/z* peaks at 987.59, 526.37, and 703.47 are assigned to the molecular weight of [Ni(SB^2^–H)_2_]·3H_2_O, [Ni(SB^3^–H)(OH)(H_2_O)], and [Ni(SB^5^–H)(OH)(H_2_O)] complexes, respectively. Besides, the peak with the highest intensity (100% abundance) that appeared at *m/z* 685.01 (Fig. S18) could be attributed to the corresponding fragment [Ni(SB^5^–H)(OH)]^+^ with one water molecule less than its precursor. Moreover, the peak corresponding to the Ni isotope appeared clearly at *m/z* of ~ 59 in the mass spectra of the studied complexes (Figs. 6, S17, and S18). Similarly, the molecular ion peaks are well in agreement with the molecular weight of the synthesized ligands (SB^1^ and SB^2^). Clearly, two molecular ion peaks of nearly equal intensity appeared at m/z of 354 and 356 for SB^1^ and m/z of 437 and 439 for SB^2^ respectively (Figs. S16 and 3). These twin molecular ion peaks represent [M − 1]^+^ and [M + 1]^+^ and appear due to bromine isotopes (^79^Br and ^81^Br)^38^. In addition, the base peak (100% intensity) of SB^1^ at m/z ~ 80 corresponds to the [SO_2_NH_2_] fragment of the sulfonamide group^39^. Also, the twin peaks that appeared at *m/z* 274 and 276 in the mass spectra of SB^1^ and SB^2^ is manifestly defining the cation [C_13_H_9_NOBr]^+^ (Fig. 7) which results from the elimination of the sulfonamide or sulfathiazole fragments in that order. The structural mass fragmentation pathways of SB^2^ ligand and [Ni(SB^3^–H)(OH)(H_2_O)] complex are represented in Figs. 7 and 8.

### Electronic spectra, magnetic, and conductivity measurements

**Figure 9:**
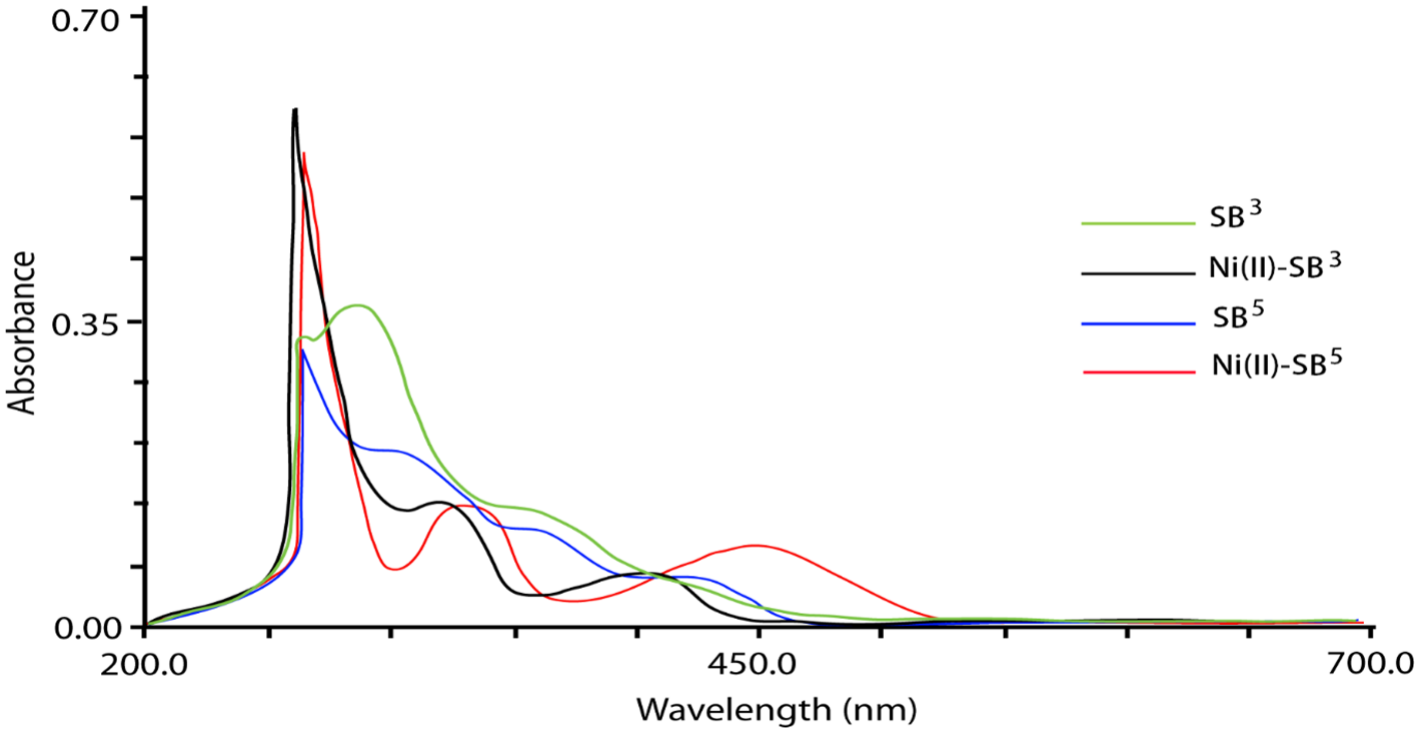
UV–Vis absorption band of SB^3^ and SB^5^ ligands and their complexes.

UV–Vis absorption spectra of the synthesized compounds were measured in the range 200–700 nm in DMF solvent. Two distinct absorption bands were observed within the ranges 265–292 and 326–367 nm attributed to the π → π* and n → π* transitions, respectively (Fig. 9). These bands are owing to the presence of OH, azomethine, and sulfonyl groups in the vicinity of the conjugated system. Similarly, these bands have appeared in the spectra of the Ni(II) complexes in the same wavelength ranges whereas obvious broadband in the region 415–454 nm is ascribed to the charge transfer of the type LMCT. This broad CT band in the absorbance spectra of the complexes completely obscured the expected d-d transitions in this region^40^.

The synthesized complexes possess a neutral non-electrolytic feature. This was verified by the low measured molar conductance of *Λ*_m_ < 4.5 Ω^–1^ mol^−1^ cm^2^ for a concentration of 10^–3^ M in DMF^39^. Additionally, different geometries of Ni (II) complexes were confirmed by the effective magnetic moments (*μ*_eff_) at room temperature (Table 1). Commonly, the diamagnetic behavior coincides with square planar geometry as in the case of [Ni(SB^2^–H)_2_]·3H_2_O complex^8^. Also, [Ni(SB^1^–H)_2_(SB^1^)]·2H_2_O that exhibited magnetic moments of 3.13 was assigned to octahedral geometry and consistent with the 1:3 (M:L) stoichiometry^41^. However, the relatively low spin values within the range 0.5–2.91 B.M of the Ni(II) complexes derived from SB^3^, SB^4^, and SB^5^ may be caused either by spin–spin interaction or tetrahedral ↔ square planar configurational equilibrium^42^. The tetrahedral percentage can be calculated by the following mathematical formula:

$${N}_{t}= \frac{{(\mu }_{obs }{)}^{2}}{({3.3)}^{2}} \times 100$$

where *N*_t_ is the tetrahedral percentage in the solid-state of the 4-coordinate complex and *μ*_obs_ is the measured magnetic moment at 296 K^43^. The calculated *N*_t_ for [Ni(SB^3^–H)(OH)(H_2_O)] was found to be 2.3% which indicates the dominance of the square planar configuration. Nevertheless, *N*_t_ values for [Ni(SB^4^–H)_2_]·4H_2_O and [Ni(SB^5^–H)(OH)(H_2_O)] were 77.8 and 42.4%, respectively.

### Thermal analysis

**Figure 10:**
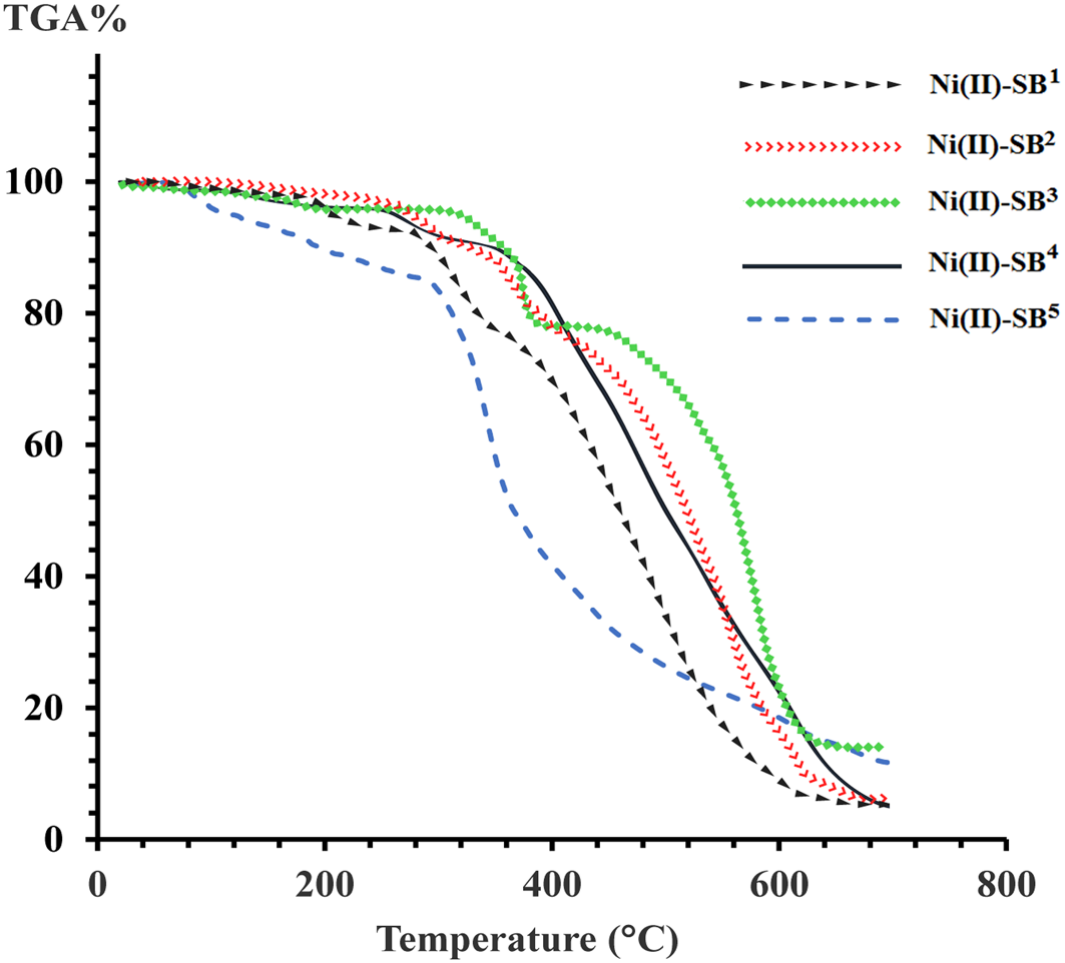
TGA of the Ni(II)-Schiff base complexes under N_2_ atmosphere.

The thermal stability of the synthesized nickel complexes was studied by TGA and DTA approaches in the temperature range up to 700 °C in a nitrogen atmosphere, Table 2 and Figs. 10 & S19. The main thermal decomposition stage of breaking the metal–ligand bonds starts in the temperature range 299.1–423.9 °C implying their thermal stability. This stage is preceded by the removal of small molecules such as hydrated H_2_O, coordinated H_2_O, NH_3_, or SO_2_. As an example, the thermal breakdown of [Ni(SB^4^–H)_2_]·4H_2_O complex proceeds in three successive steps. The first step with a percentage mass loss of 6.32% (calc. 6.20%) was assigned to the removal of four outer sphere water molecules. This step was followed by the elimination of inner sphere small molecules (2 NH_3_ and 2 SO_2_) which started at the temperature of 220.4 °C. The decomposition of the bulk of two ligand molecules (C_32_H_14_N_4_OS_2_Br_4_) begins at 347.0 °C with a DTA peak maximum (*T*_max_) at 531.1 °C. The elevated *T*_max_ for this step is an indication of the complex thermal stability^44^. For all complexes, the thermal decomposition ended with the formation of NiO residue with a mass percentage in the range of 6.42% to 14.18%. Noteworthy, the combination of the decomposed fragments is in accord with the proposed structure of a given complex as deduced from spectroscopic and analytical data. However, the common overlap in the steps of the thermal decomposition could explain the slight variation between the calculated and found masses of the yielded thermal fragments.

**Table 2:** Thermogravimetric decomposition steps of Ni(II) complexes derived from SB^1^–SB^5^.

Complex (M:L)	*T*_*max*_ °C	Temp. Range (^o^C)	Wt. loss / Residue %		Assignment
			Found	Calc	
**[Ni(SB**^**1**^**)**_**2**_**(SB**^**1**^–**H)**]·**2H**_**2**_**O** (1:3)	132.6	31.0–230.0	5.86	6.05	–2H_2_O, –2NH_3_
	313.9	230.0–377.4	16.43	16.59	–3SO_2_
	436.8	377.5–695.8	71.28	70.91	–C_39_H_25_O_2_N_4_Br_3_
		Residue	6.43	6.45	NiO
**[Ni(SB**^**2**^**)**_**2**_]·**3H**_**2**_**O** (1:2)	185.4	34.9–265.4	9.24	8.92	–3H_2_O, –2NH_3_
	305.4	265.4–389.2	12.71	12.98	–2SO_2_
	446.2	389.2–697.8	70.58	70.53	–C_32_H_16_N_4_OS_2_Br_2_
		Residue	7.47	7.56	NiO
**[Ni(SB**^**3**^–**H)(OH)(H**_**2**_**O)]** (1:1)	143.6	35.3–201.3	6.79	6.84	–2H_2_O
	374.5	304.6–423.9	15.52	15.39	–NH_3_, –SO_2_
	517.5	423.9–698.6	63.78	63.59	–C_13_H_5_NBr_2_
		Residue	13.91	14.18	NiO
**[Ni(SB**^**4**^–**H)**_**2**_]·**4H**_**2**_**O** (1:2)	151.9	28.3–220.4	6.32	6.20	–4H_2_O
	320.1	220.4–347.0	13.96	13.94	–2NH_3_, –2SO_2_
	531.1	347.0–698.3	73.32	73.44	–C_32_H_14_N_4_OS_2_Br_4_
		Residue	6.40	6.42	NiO
**[Ni(SB**^**5**^–**H)(OH)(H**_**2**_**O)]** (1:1)	96.6	39.7–160.3	5.04	5.12	–2H_2_O
	236.4	160.3–299.1	11.36	11.52	–NH_3_, –SO_2_
	327.2	299.1–698.6	73.20	72.75	–C_16_H_6_N_2_SI_2_
		Residue	10.40	10.61	NiO

### Molecular modeling study

#### Structural optimization and reactivity descriptors

**Figure 11:**
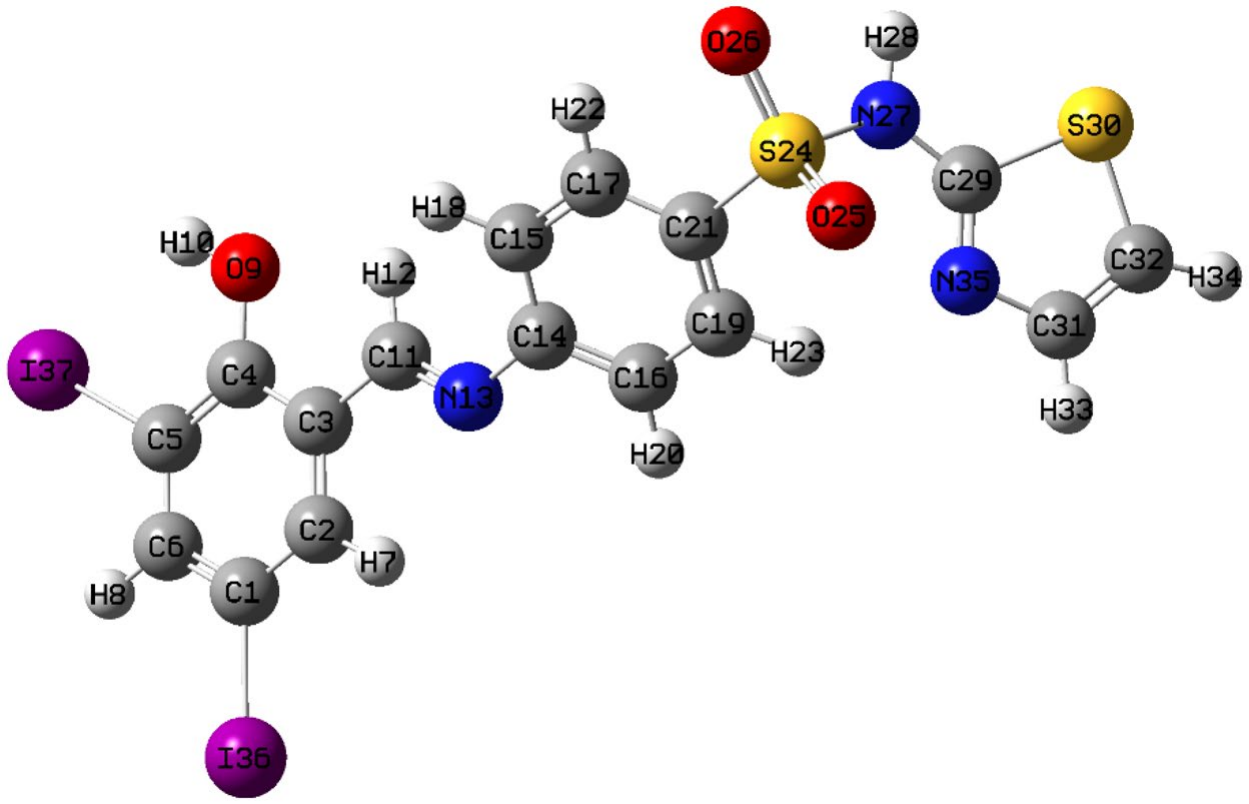
The optimized structure of Schiff base sulfathiazole, 5-(2-hydroxy-3,5-diiodobenzylidene amino)-*N*-(1,3-thiazol-2-yl)benzenesulfonamide (SB^5^) using DFT-B3LYP/ Lanl2dz method by GAUSSIAN 09 software version 9.5 and GAUSSVIEW 6.0.16.

**Figure 12:**
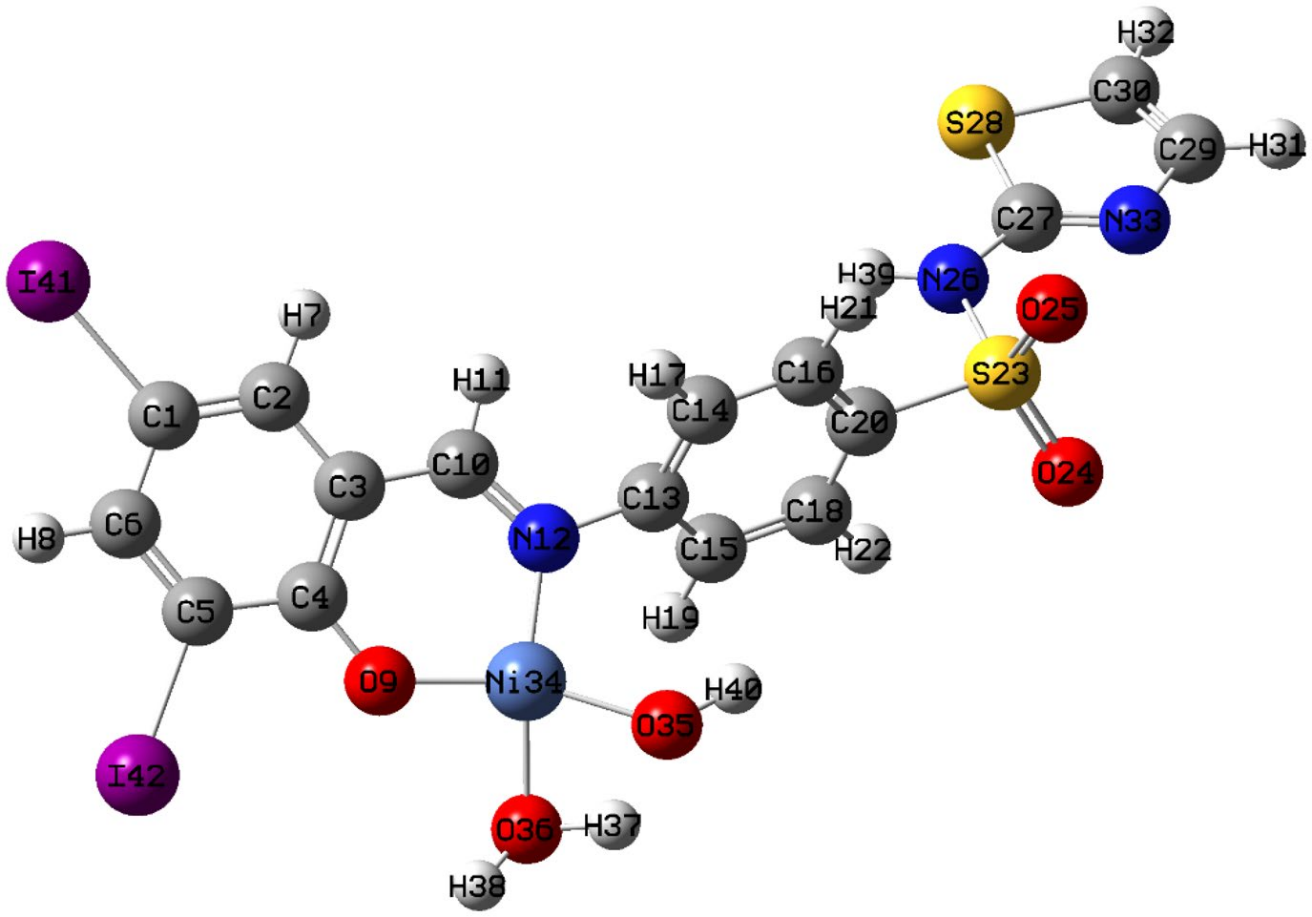
The optimized structure of [Ni(SB^5^–H)(OH)(H_2_O)] complex using DFT-B3LYP Lanl2dz method by GAUSSIAN 09 software version 9.5 and GAUSSVIEW 6.0.16.

**Figure 13:**
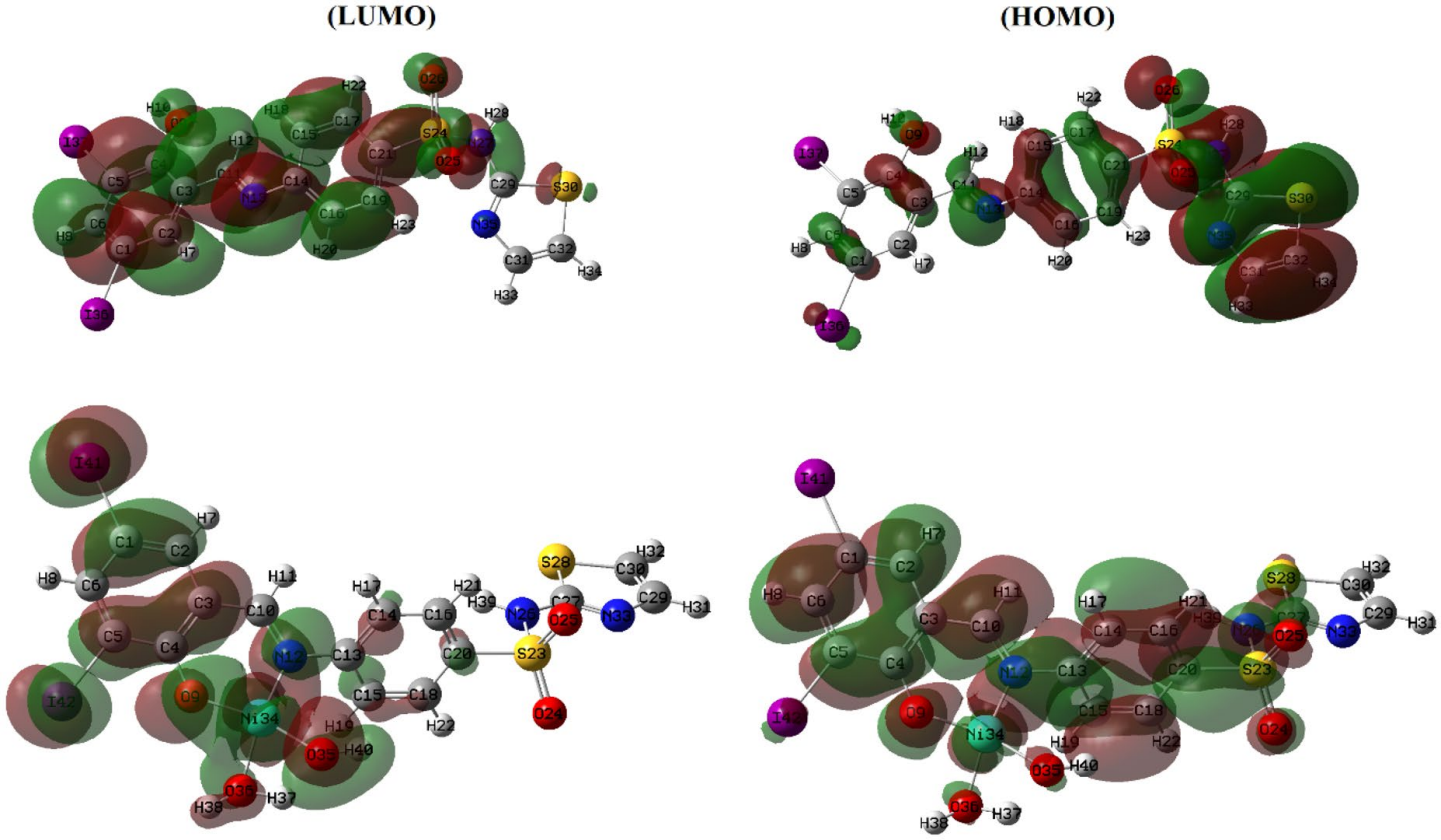
LUMO and HOMO of SB^5^ and [Ni(SB^5^–H)(OH)(H_2_O)] complex, using GAUSSIAN 09 software version 9.5 and GAUSSVIEW 6.0.16.

**Figure 14:**
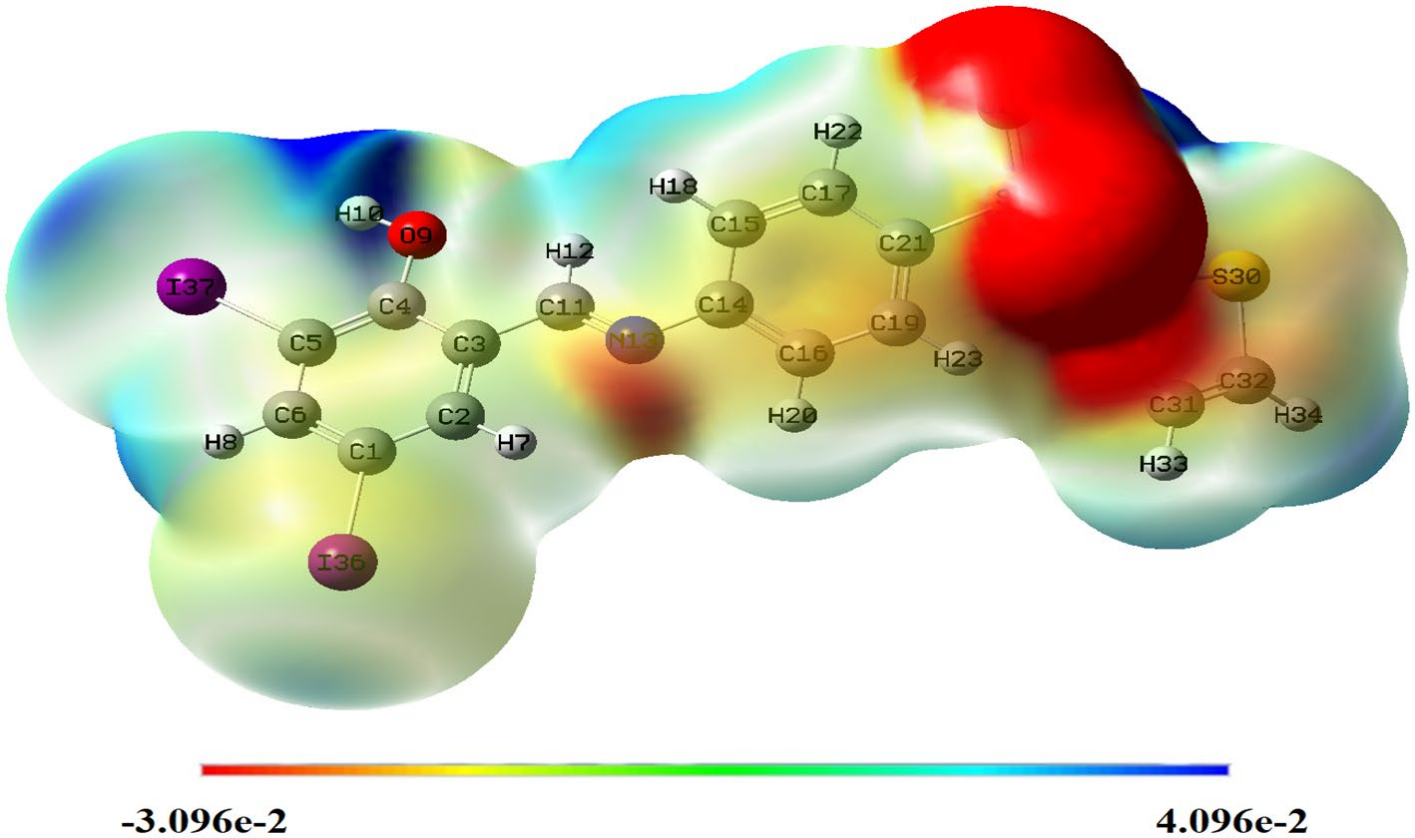
Molecular electrostatic potential map of SB^5^, using GAUSSIAN 09 software version 9.5 and GAUSSVIEW 6.0.16.

Molecular parameters of all synthesized ligands and Ni(II) complex of SB^5^ including frontier eigenvalues, dipole moments, bond lengths, and angles were calculated using DFT with B3LYP function and Lanl2dz basis set (Table 3 and Figs. 11, 12 & S20–S25). The chemical reactivity of the synthesized Schiff bases was predicted from the energy difference between the low unoccupied (LUMO) and the high occupied molecular orbitals (HOMO) of the corresponding ligand, *E* (Fig. 13)^8^. Noticeably, there is a good correlation between the structure of synthesized ligands and their reactivity. For example, the sulfathiazole Schiff bases, SB^2^, and SB^4^ showed nearly similar *E* values within the range 3.466–3.487 eV. Also, the sulfonamide Schiff bases, SB^1^ and SB^3^ exhibited analogous *E* of 3.553–3.611 eV which implies parallel reactivity of these Schiff bases and indicates the existence of structural-activity relation among the ligands. Based on the optimization parameters, the halogen substituents induce extra stability of the LUMO levels^45^. This can be predicted by the higher negative total energy, *E*_T_, of the di-halogenated ligands (SB^3^, SB^4^, and SB^5^) compared to the mono-halogenated ligands (SB^1^ and SB^2^) (Table 3). Moreover, the iodide substituents gave more induced LUMO level stability than the bromide substituents due to the relatively large size of iodide and hence increases the electrophilic character^45^. Furthermore, the high negative total energy of the complex [Ni(SB^5^–H)(OH)(H_2_O)], *E*_T_ = –1370 Hartree, indicates the great stability of the isolated complex compared to its free ligand^46^. Besides, the distortion of the standard square planar geometry of the complex Ni(II)–SB^5^ (1:1) was estimated from the calculated bond angles around the Ni(II) core which are consistent with the determined tetrahedral percentage (*N*_t =_ 42.4%) from the experimental magnetism value. The estimated angles N(12)–Ni(34)–O(9), O(9)–Ni(34)–O(36), O(36)–Ni(34)–O(35), and O(35)–Ni(34)–N(12) of Ni(II)–SB^5^ complex were 95.38°, 87.47°, 78.36°, and 98.97°, respectively representing a mixture of square planar and tetrahedral geometries around the central Ni (II) ion (Table S2 and Fig. 14). Additionally, it was observed that the bonds between the atoms incorporated in the coordination sphere (C(4)–O(9) and C(10)=N(12)) showed longer lengths in the complex than in the free ligand (Table S2)^47^. However, no change in the bond lengths was noted in the sulfonamide or sulfathiazole parts after the complex formation excluding them from any expected coordination. The magnitude of the calculated dipole moment (D) gives a good indication of the capability of the compound to penetrate the biological cell membrane. Generally, the lower the dipole moment, the higher the penetration strength across the phospholipid bilayer^48^. In addition, based on the other calculated molecular descriptors; softness (*S*), hardness (*η*), electrophilicity (*ω*), and electronegativity (*χ*), the estimated chemical reactivity of the ligands toward metal ions or any neighboring biological receptors is in the order: SB^5^ >  SB^4^ >  SB^2^ >  SB^3^ >  SB^1^, where ^4^ SB^5^ is proposed to have the highest softness character (*S* = 0.300 eV^−1^) and the smallest *E,* i.e. the highest electron donation ability.

**Table 3:** The molecular parameters of Schiff bases and the Ni(II)–SB^5^ complex calculated by DFT-B3LYP/Lanl2dz method.

Compound	*E*_T_ (Hartree)	*D* (Debye)	*E*_HOMO_ (eV)	*E*_LUMO_ (eV)	Δ*E* (eV)	*η* (eV)	*S* (eV^–1^)	*μ* (eV)	*χ* (eV)	*ω* (eV)
SB^1^	–860	4.640	–6.533	–2.922	3.611	1.806	0.277	–4.728	4.728	6.189
SB^2^	–1040	4.252	–6.604	–3.117	3.487	1.744	0.287	–4.861	4.861	6.774
SB^3^	–873	5.255	–6.657	–3.104	3.553	1.777	0.281	–4.881	4.881	6.703
SB^4^	–1052	4.396	–6.722	–3.256	3.466	1.733	0.289	–4.989	4.989	7.181
**SB**^**5**^	–1049	4.452	–6.557	–3.220	3.337	1.669	0.300	–4.889	4.889	7.161
**[Ni(SB**^**5**^–**H)(OH)(H**_**2**_**O)]**	–1370	3.149	–6.068	–3.014	3.054	1.527	0.327	–4.541	4.541	6.752

Furthermore, the electron density cloud was mapped over the surface of the ligands to evaluate the nucleophilic (high electron density, red) and the electrophilic (low electron density, blue) sites of interactions (Figs. 14 and S25)^49^. The regions with zero electronic potential are denoted by green. For all ligands, the (SO_2_) group represents the site with intense electron density, while there is a lack of electron density at the hydrogen terminals. Nevertheless, the N(13) of the azomethine and O(9) of the phenol parts of SB^5^ (Fig. 14) showed moderate nucleophilicity with Mulliken charges of − 0.587 and − 0.596 a.u. for N(13) and O(9) respectively, and hence can be possible electron donor atoms in the coordination mode as proposed spectroscopically.

#### Molecular docking

**Figure 15:**
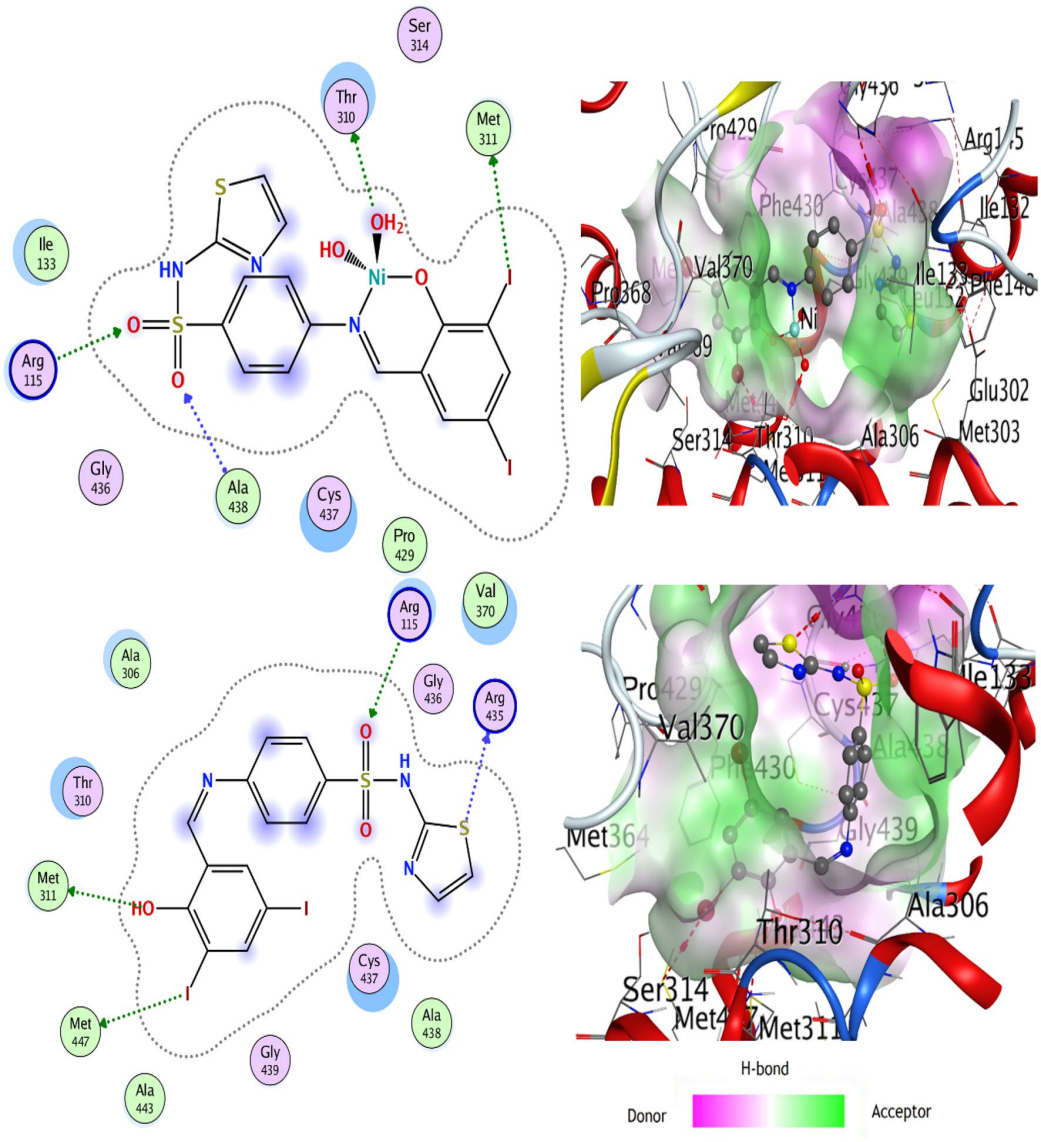
Ligand-receptor interactions of SB^5^ and its Ni(II) complex versus *3s7s* protein, using the Molecular Operating Environmental module (MOE 2015.10) software.

Docking investigation is an essential step preceding the in vitro study of any proposed biologically active compound. This approach elucidates the ligand-receptor site and type of interactions. It also gives an estimation of the distance between the ligand and the receptor inside the interaction grid. The scoring energy of each pose simulated by the docking calculations reflects the degree of inhibition effect of the corresponding ligand. In the present study, the selected protein *3s7s* represents the crystal structure of the human placental aromatase enzyme that catalyzes the synthesis of estrogen hormone and contributes to estrogen-dependent breast cancer^50^. All ligands possess an appreciable extent of interactions with the receptor protein based on the scoring energy (*S*), the number, and the length of H-bonds (Table 4 and Figs. 15 & S26). Favorable interaction was discerned for SB^5^ with many bonds formed inside the protein binding pocket and high scoring energy of (− 8.6219 kcal/mol). The great number of H-bonds would successfully facilitate its penetration across the cell membrane.

**Table 4:** The interaction parameters of the synthesized compounds versus *3s7s* protein.

Compound	Ligand site	Receptor site	Interaction type	Distance (Å)	*E* (kcal/mol)	*S* (kcal/mol)
**SB**^**1**^	N 28	PHE 430	H-donor	3.28	− 0.7	− 6.6717
	N 28	ARG 435	H-donor	3.33	− 1.2	
	Br 31	MET 447	Halogen bonding	4.31	− 0.4	
	O 27	ARG 115	H-acceptor	2.81	− 1.8	
**SB**^**2**^	S 31	MET 303	H-donor	3.94	− 0.2	− 8.3594
	Br 37	ARG 435	Halogen bonding	3.32	− 0.8	
	O 27	ALA 307	H-acceptor	3.20	− 0.8	
**SB**^**3**^	O 9	MET 311	H-donor	3.17	− 1.9	− 7.1499
	N 27	GLU 302	H-donor	3.28	− 1.4	
	N 27	MET 303	H-donor	3.70	− 3.1	
	Br 30	PRO 429	Halogen bonding	3.04	− 0.5	
	Br 31	MET 447	Halogen bonding	3.52	− 0.4	
	6-ring	ALA 306	π-H	3.73	− 0.7	
**SB**^**4**^	O 9	ARG 435	H-donor	2.99	− 2.2	− 8.9932
	Br 37	ARG 375	Halogen bonding	3.31	− 1.0	
**SB**^**5**^	O 9	MET 311	H-donor	3.40	− 1.2	− 8.6219
	S 30	ARG 435	H-donor	3.87	− 0.9	
	I 37	MET 447	Halogen bonding	3.99	− 0.8	
	O 26	ARG 115	H-acceptor	3.50	− 0.6	
**[Ni( SB**^**5**^**-H)(OH)(H**_**2**_**O)]**	O 9	MET 311	H-donor	3.76	− 1.1	− 8.5098
	I 37	MET 447	Halogen bonding	4.10	− 1.0	
**Sulfamethoxazole moiety of standard drug**	N 15	LEU 372	H-donor	3.24	− 1.1	− 6.2313
	O 13	MET 374	H-acceptor	3.08	–1.6

Also, the ligands SB^4^ and SB^2^ exhibited reasonable interaction with the target protein with scoring energies of − 8.9932 and − 8.3594 kcal/mol, respectively, indicating their high binding affinity with the receptor protein^51^. Effectively short H-bond was observed in most of the docked protein–ligand complexes. As an example, the interaction of SB^1^ and SB^4^ through O(27) with ARG 115 amino acid and O(9) with ARG 435 amino acid respectively, displayed bond lengths that are less than 3.5 Å^8,48^. Worth mentioning that the negative values of the binding energies (Table 4) indicate spontaneous interaction with the protein. Moreover, a number of halogen bonds were observed between ligands and some receptor sites inside the protein pocket (Figs. 15 and S26). However, these multiple weak bonds boost the inhibition impact of the ligands. Docking parameters against the protein *3s7s* were also evaluated for a standard sulfa drug Trimethoprim-sulfamethoxazole (Bactrim). The drug contains a sulfamethoxazole moiety that resembles the synthesized ligands. The drug showed less scoring energy, Table 4 and Fig. S27, than the synthesized Schiff bases, implying high *3s7s* inhibition activity for the ligands SB^1^- SB^5^.

### Biological activity assessment

#### Antimicrobial potency study

**Figure 16:**
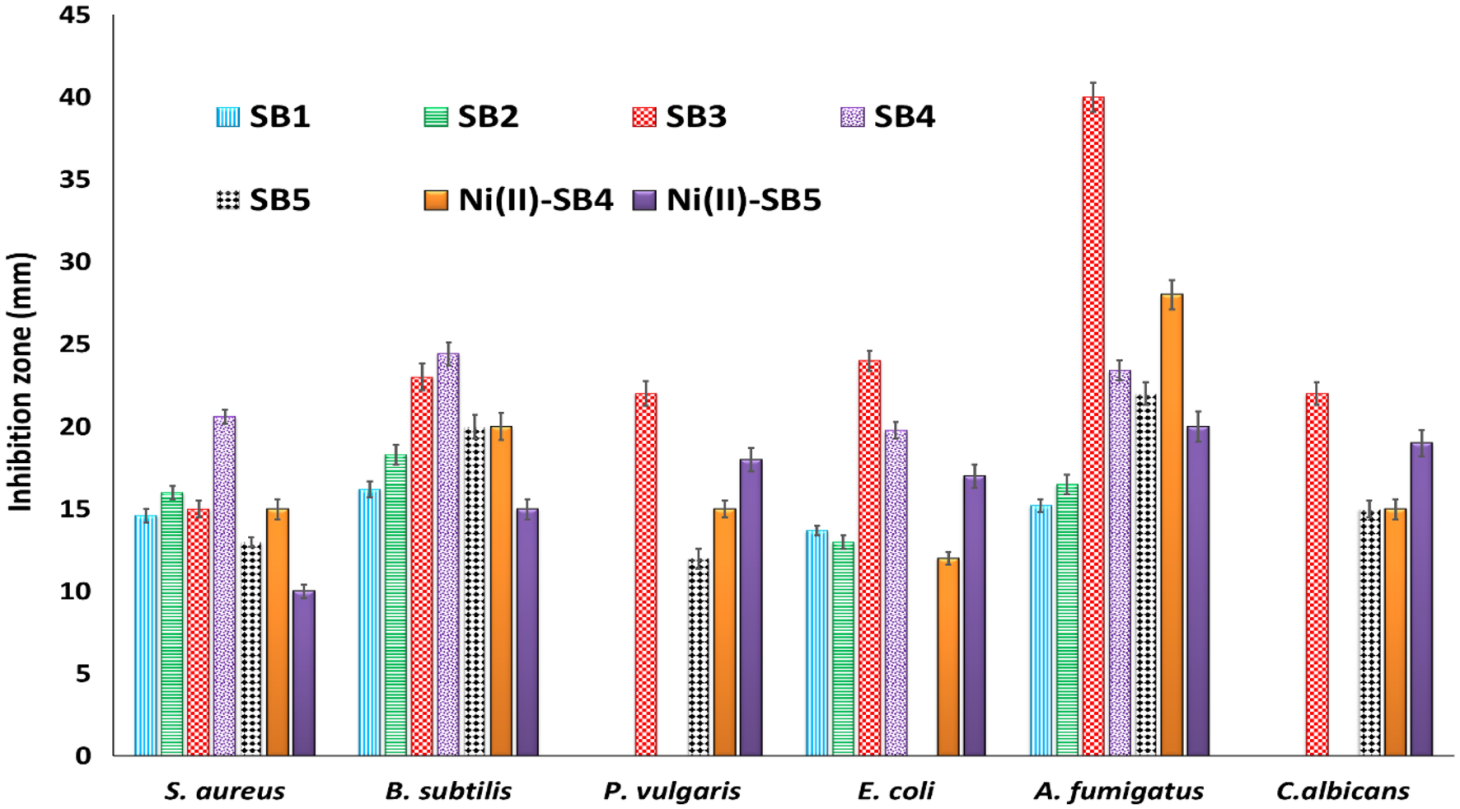
Inhibition zone (mm) of the synthesized compounds against selected microbes.

The biological activity data of all ligands (SB^1^–SB^5^) and some selected complexes are summarized in Table 5 and Fig. 16. The observed mean zone diameter of inhibition in mm is considered a good index of the antimicrobial activity of the tested samples. For most of the studied strains, ligands show more inhibition efficacy than their corresponding complexes. However, a plausible comparative study of the antimicrobial potency of the compounds under investigation was achieved based on their minimum inhibitory concentration values in μM rather than µg/ml owing to their different molecular weights and according to the microdilution broth assay. Among the ligands, SB^4^ showed superior broad-spectrum activity in prohibiting microbial growth that is sometimes matching or even better than that of the utilized references. For instance, *B. subtilis*, *E. coli*, and *A. fumigatus* strains were very sensitive to SB^4^ with MIC values of 0.46, 7.54, and 0.95 µM, respectively, which are lower than that displayed by ampicillin (1.40 μM), gentamycin (8.38 μM), and ketoconazole (1.88 μM) as reference controls. In general, it is difficult to deduce a certain conclusion relating the observed activity with the skeleton of such compounds that contain diverse bioactive moieties such as benzenesulfonamide or salicylaldehyde imine including hydroxyl group, halogens, or heteroatomic thiazole ring, (Fig. 1). Notwithstanding such difficulty, the superior potency of the Schiff base ligand (SB^4^) could be credited to the consortium of different bioactive substituents as two bromine atoms and a thiazole ring attached to the Schiff base backbone structure. The two bromine substituents probably enhance the inhibitory effect of the attached system by their electron-withdrawing property as reported for analogous Schiff base compounds^52,53^. This is in harmony with the spotted decrease in the activity of SB^1^ or SB^2^ (mono-brominated Schiff bases), and SB^3^ (absence of thiazole ring) in comparison with SB^4^. Also, the substituent size (I or Br) is a crucial factor in determining the feasibility of pathogen inhibition^54,55^. Noticeably, the Gram-negative *P. vulgaris* bacteria showed the least susceptible character against most tested compounds (Table 5) pointing to the importance of its outer lipid membrane as an additional shield despite its thinner peptidoglycan wall^56^.

**Table 5:** Antibacterial and antifungal inhibition zone in mm and the MIC (µM) of the Schiff bases and some of their complexes. NA: no activity, NT: not tested.

Compound	Gram-positive bacteria		Gram-negative bacteria		Fungi	
	*S. aureus*	*B. subtilis*	*P. vulgaris*	*E. coli*	*A. fumigatus*	*C. albicans*
**SB**^**1**^	14.6 ± 0.4 (351.9)	16.2 ± 0.5 (176.0)	NA	13.7 ± 0.3 (> 1000)	15.2 ± 0.4 (176.0)	NA
**SB**^**2**^	16.0 ± 0.4 (142.6)	18.3 ± 0.6 (35.7)	NA	13.0 ± 0.4 (> 1000)	16.5 ± 0.6 (142.6)	NA
**SB**^**3**^	15.0 ± 0.5 (> 1000)	23.0 ± 0.8 (719.9)	22.0 ± 0.77 (> 1000)	24.0 ± 0.6 (> 1000)	40.0 ± 0.9 (359.9)	22.0 ± 0.7 (719.9)
**SB**^**4**^	20.6 ± 0.4 (7.54)	24.4 ± 0.7 (0.46)	NA	19.8 ± 0.5 (7.54)	23.4 ± 0.6 (0.95)	NA
**SB**^**5**^	13.0 ± 0.3 (> 1000)	20.0 ± 0.7 (511.3)	12.0 ± 0.6 (> 1000)	NA	22.0 ± 0.7 (511.3)	15.0 ± 0.5 (255.6)
**[Ni(SB**^**4**^**–H)**_**2**_**]·4H**_**2**_**O**	15.0 ± 0.6 (> 1000)	20.0 ± 0.8 (537.3)	15.0 ± 0.5 (> 1000)	12 0.0 ± 0.4 (> 1000)	28.0 ± 0.9 (537.3)	15.0 ± 0.6 (268.7)
**[Ni(SB**^**5**^**–H)(OH)(H**_**2**_**O)]**	10.0 ± 0.4 (> 1000)	15.0 ± 0.6 (88.8)	18.0 ± 0.7 (> 1000)	17.0 ± 0.7 (88.8)	20.0 ± 0.9 (> 1000)	19.0 ± 0.8 (> 1000)
**Ampicillin**	23.7 ± 0.6 (2.85)	26.4 ± 0.5 (1.40)	NT	NT	NT	NT
**Gentamycin**	NT	NT	25.0 ± 0.3 (8.38)	30.0 ± 0.4 (8.38)	NT	NT
**Ketoconazole**	NT	NT	NT	NT	17.1 ± 0.3 (1.88)	20.2 ± 0.4 (1.88)

#### The antiproliferative potency

**Figure 17:**
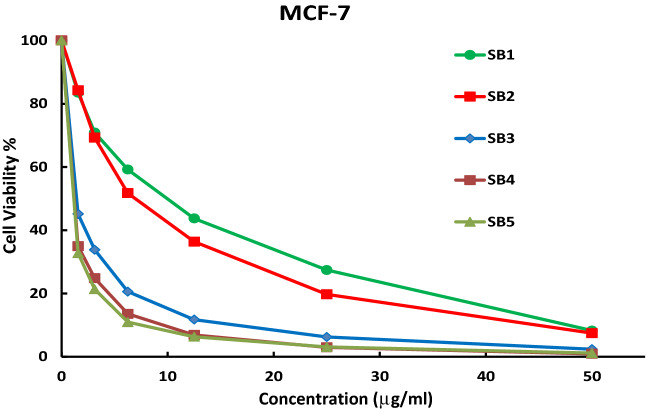
Inhibitory activity of the synthesized ligands against breast carcinoma cells MCF-7.

The concentration of some selected synthesized compounds as anticancer candidates to inhibit the growth of the proliferative cells by 50% (IC_50_) in μM was evaluated against MCF-7 (human breast cancer cell line). All ligands and some of their nickel complexes showed appreciable cytotoxicity against MCF-7 (Table 6 and Figs. 17 & S28), compared to cisplatin as a chemotherapy standard drug for breast cancer. Based on the IC_50_ values, both SB^2^ (16.0 μM) and SB^4^ (18.8 μM) have nearly equipotent antiproliferative activity as cisplatin (19.0 μM) against MCF-7 cells and both are more active than SB^1^ (28.2 μM) and SB^3^ (29.7 μM) (Table 6). This variety in IC_50_ values points to the importance of the existence of the thiazole moiety and Br atom(s) in the building structure of the Schiff base ligands (SB^2^ and SB^4^) (Fig. 1) as deduced in the antimicrobial Sect. ^8,55^. Interestingly, SB^5^ (6.32 μM) exhibited threefold inhibition efficacy more than cisplatin recommending it as a promising breast anticancer drug and in accord with US NCI program (IC_50_ < 10 μM)^57^. A similar cytotoxicity tendency was observed for complexes where [Ni(SB^2^–H)_2_]·3H_2_O (11.2 μM) and [Ni(SB^4^–H)_2_]·4H_2_O (4.33 μM) have nearly twofold or fourfold potency in comparison with cisplatin against the human MCF-7 cell lines. The double cytotoxicity of Ni(SB^4^–H)_2_]·4H_2_O could be related to the presence of two additional Br atoms than the case of [Ni(SB^2^–H)_2_]·3H_2_O complex as both complexes have the same 4-coordinate geometry, metal to ligand ratio (1:2), and the same bioactive functional groups (benzenesulfonamide, thiazole, and salicylaldehyde imine). Unlike the SB^5^ ligand, [Ni(SB^5^–H)(OH)(H_2_O)] complex (1:1) has insignificant activity against MCF-7 cells where the chelation suppresses the antiproliferation in this case as discerned from the docking simulation.

**Table 6:** Cytotoxicity activity (IC_50_) in μg/ml and µM of Schiff bases and some of their complexes.

Compounds	MCF-7		OEC
	μg/ml	μM	μg/ml
**SB**^**1**^	10.0 ± 0.9	28.2 ± 1.9	NT
**SB**^**2**^	7.0 ± 0.6	16.0 ± 1.2	NT
**SB**^**3**^	12.9 ± 1.2	29.7 ± 1.8	NT
**SB**^**4**^	9.73 ± 1.0	18.8 ± 1.3	49.53 ± 1.95
**SB**^**5**^	3.86 ± 0.5	6.32 ± 0.8	NT
**[Ni( SB**^**2**^**–H)**_**2**_**]·3H**_**2**_**O**	11.3 ± 1.1	11.2 ± 0.9	62.23 ± 3.11
**[Ni( SB**^**4**^**–H)**_**2**_**]·4H**_**2**_**O**	5.04 ± 0.6	4.33 ± 0.5	33.59 ± 1.68
**[Ni( SB**^**5**^**–H)(OH)(H**_**2**_**O)]**	365 ± 24.6	> 100	> 100
**Cisplatin**	5.71 ± 0.7	19.0 ± 2.3	32.68 ± 2.74

**Figure 18:**
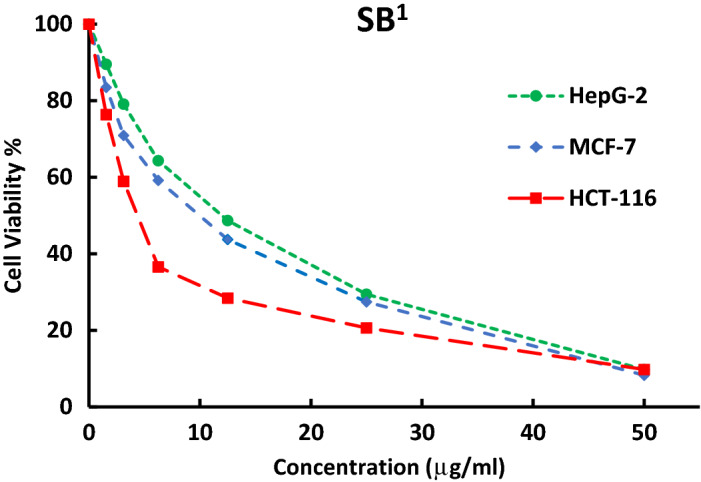
The inhibitory dose curves of SB^1^ against three cell lines HepG-2, MCF-7, and HCT-116.

**Figure 19:**
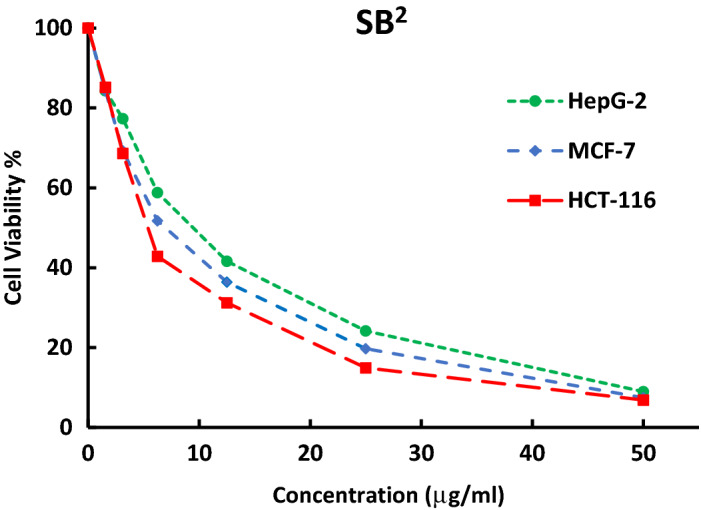
The inhibitory dose curves of SB^2^against three cell lines HepG-2, MCF-7, and HCT-116.

Furthermore, a comparative study was also made to compare the cytotoxicity of some ligands (SB^1^ and SB^2^) against different human cell lines HCT-116 (colon carcinoma), HepG-2 (hepatocellular carcinoma), and MCF-7 (breast carcinoma) (Figs. 18, 19). The investigated ligands SB^1^ and SB^2^ exhibited the same trend of cytotoxicity where the best inhibitory activity was observed against HCT-116 (IC_50_ 12.4 and 12.3 μM) and the least was against HepG-2 (IC_50_ 33.8 and 21.7 μM), respectively. Noteworthy, SB^4^ and all Ni (II) complexes exhibited less cytotoxic activity against the human normal oral epithelial cell, OEC, compared to cisplatin Table 6 and Fig. S29.

## Conclusion

In the current investigation, five new nickel complexes of halogenated sulfonamide-based Schiff bases (SB^1^–SB^5^) were synthesized and structurally explored by microanalytical analyses, FT-IR, NMR, UV–Vis., MS, and thermal analysis techniques. Spectral studies supported the bidentate coordination mode of all ligands towards the Ni(II) through the phenolic oxygen after deprotonation and the nitrogen atom of the azomethine group. The magnetic data revealed that the geometries of complexes are octahedral, square planar, or tetrahedral. The recorded molecular ion peaks (*m/z*) are in accordance with the determined formula mass from the microanalytical technique. The molecular descriptor parameters calculated by DFT-B3LYP/ Lanl2dz and the molecular docking simulation with the breast cancer protein (*3s7s*) approved the structural-activity relationship of the investigated compounds. SB^4^ showed the most significant inhibition activity towards some pathogen strains (*B. subtilis*, *E. coli*, and *A. fumigatus*) which is attributed to the presence of two electron-withdrawing bromine atoms in the salicylidene moiety and the attached thiazole ring to the bioactive benzenesulfonamide group. According to the IC_50_ values, SB^5^ and [Ni(SB^4^–H)_2_]·4H_2_O have nearly threefold or fourfold potency in comparison with cisplatin against breast carcinoma cells (MCF-7) recommending them as promising antiproliferative agents after further drug authorization processes. Ultimately, the nickel (II) complex derived from SB^4^ is economically expected to be of less expense with respect to other chemotherapeutic platinum-based drugs.

## Supplementary Information

Supplementary Information.

## Data Availability

All data generated or analyzed during this study are included in this published article and its supplementary information files.
