# Supplementary material for: Designing metal chelates of halogenated sulfonamide Schiff bases as potent nonplatinum anticancer drugs using spectroscopic, molecular docking and biological studies
Source: Sci Rep. 2022 Nov 23;12:20192. doi: 10.1038/s41598-022-24512-y (PMC9691640; doi:10.1038/s41598-022-24512-y)
Supplement: Supplementary file 1 — Supplementary Information. [file 41598_2022_24512_MOESM1_ESM.docx]

Supplementary data

**Designing metal chelates of halogenated sulfonamide Schiff bases as potent nonplatinum anticancer drugs using spectroscopic, molecular docking, and biological studies**

**Rehab M. I. Elsamra*· Mamdouh S. Masoud · Ahmed M. Ramadan**

Chemistry Department, Faculty of Science, Alexandria University, P.O. Box 426, Alexandria 21321, Egypt

^*^To corresponding authors: e-mail: [rehab_elsamra@alexu.edu.eg](mailto:rehab_elsamra@alexu.edu.eg)


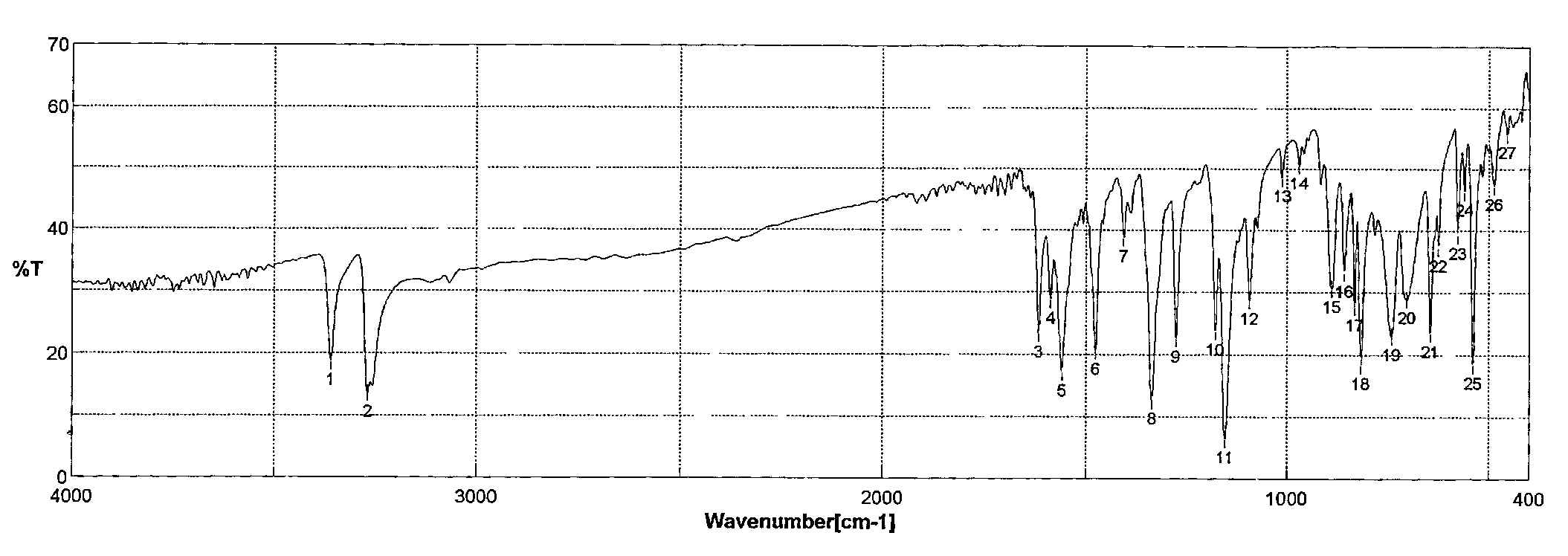


**Figure S1.** FT-IR of SB^1^ ligand

**
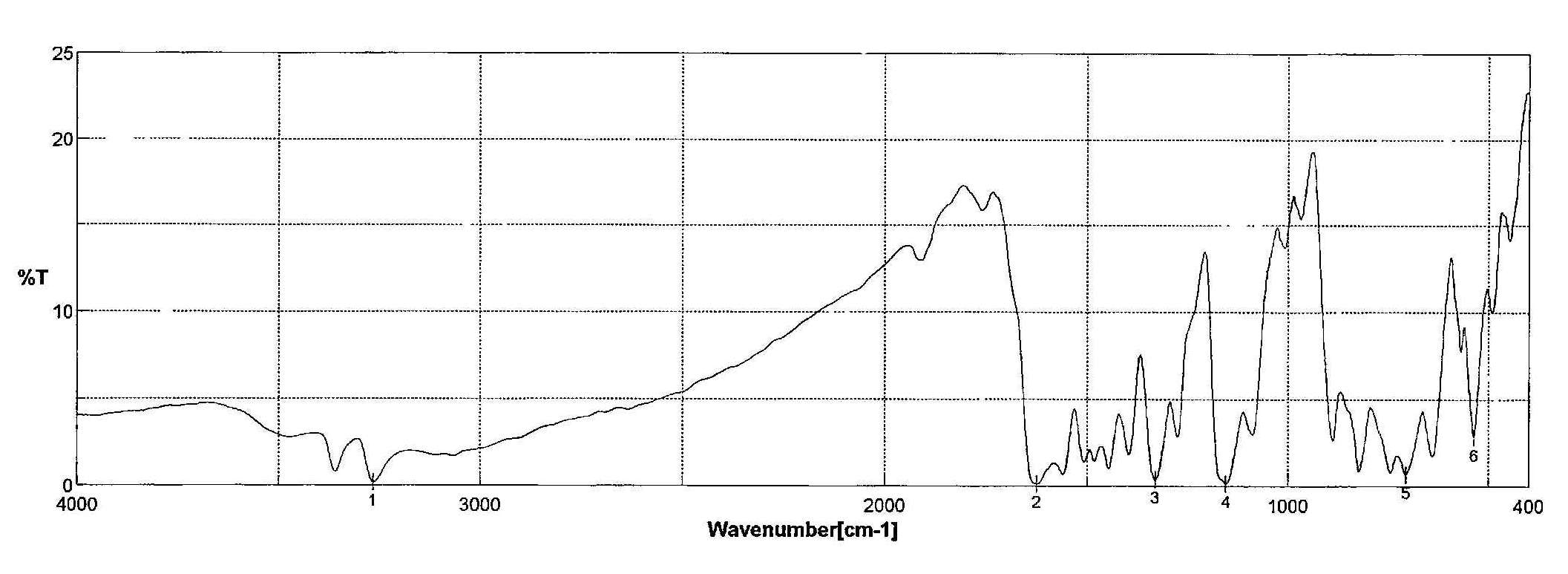
**

**Figure S2.** FT-IR of [Ni(SB^1^-H)_2_(SB^1^)].2H_2_O complex

**
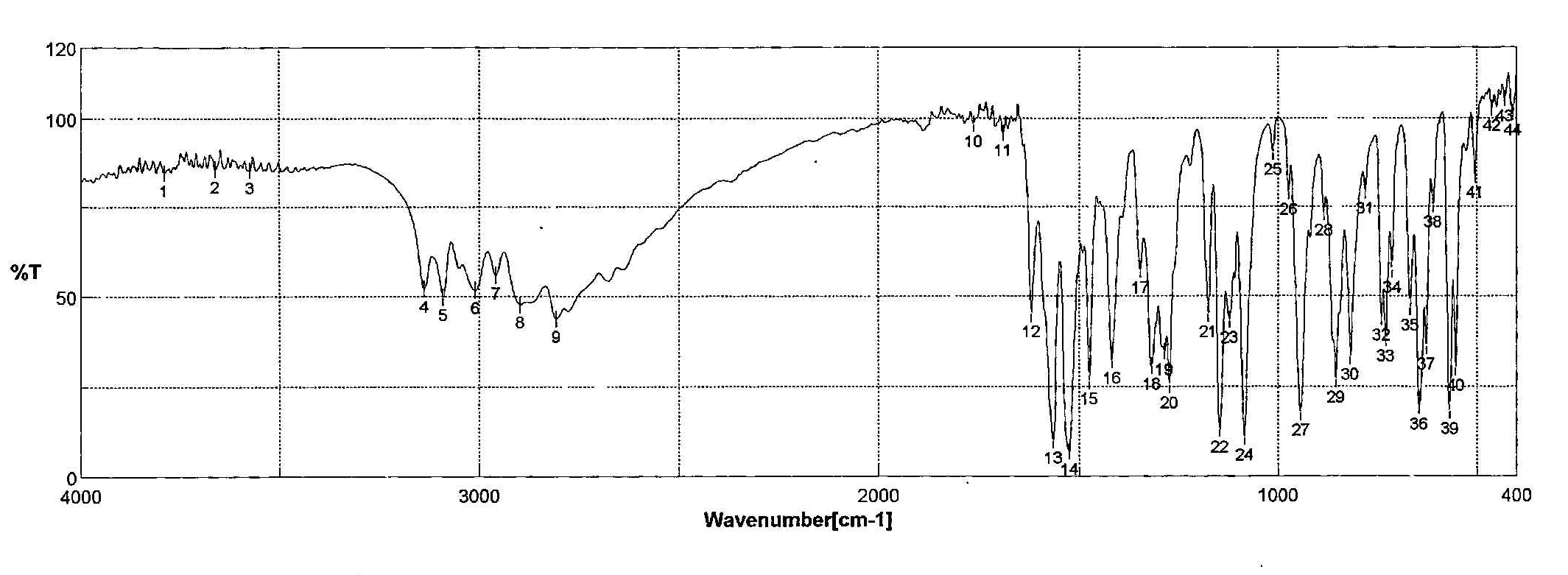
**

**Figure S3.** FT-IR of SB^2^ ligand

**
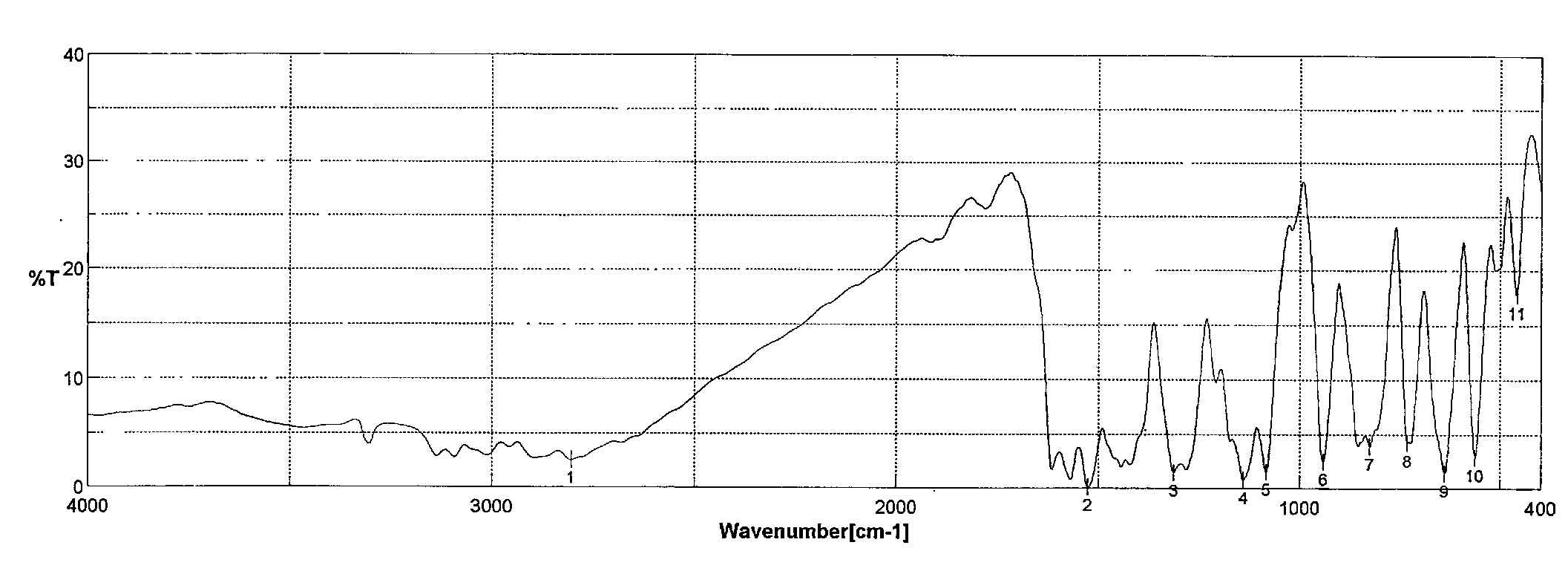
**

**Figure S4.** FT-IR of [Ni(SB^2^-H)_2_].3H_2_O complex


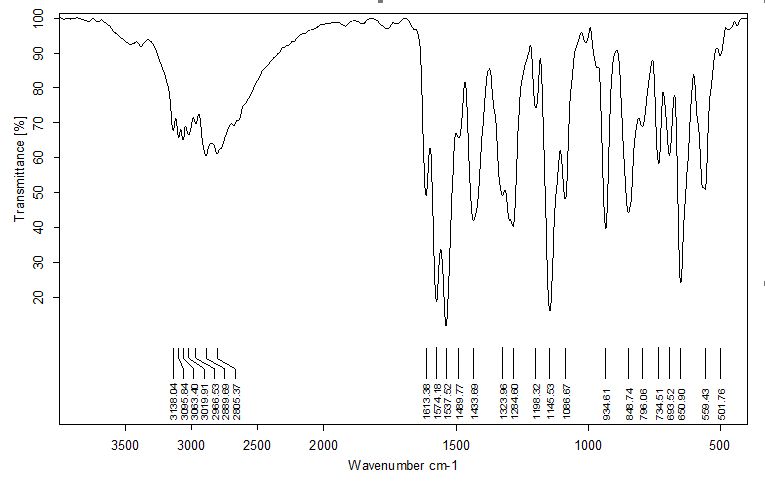


**Figure S5.** FT-IR of SB^4^ ligand


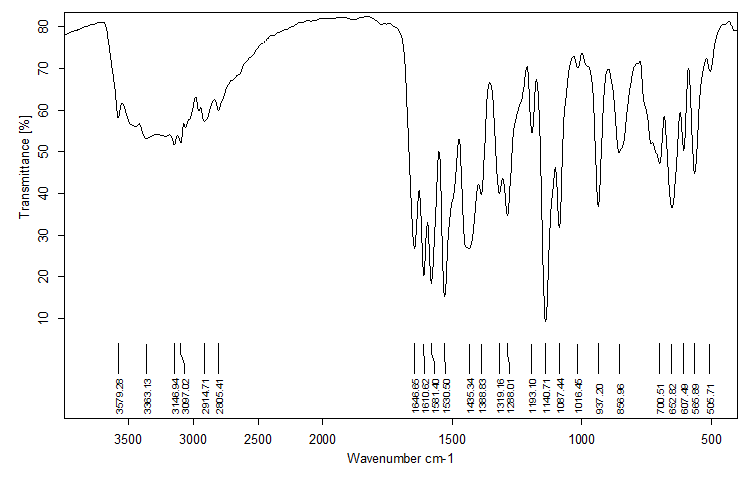


**Figure S6.** FT-IR of [Ni(SB^4^-H)_2_].4H_2_O complex

**
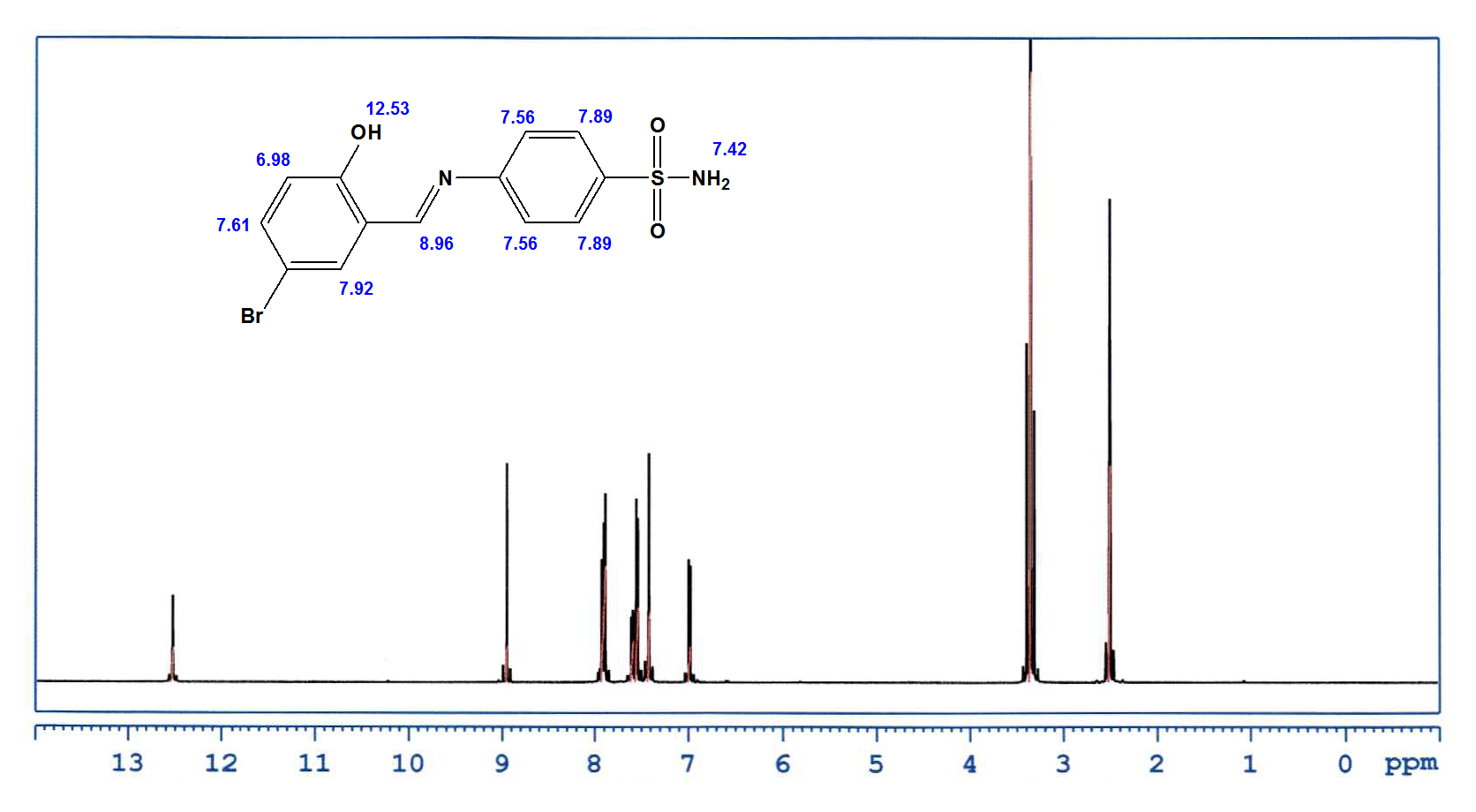
**

**Figure S7.** ^1^H NMR spectra of SB^1^ in DMSO-*d*_6_

**
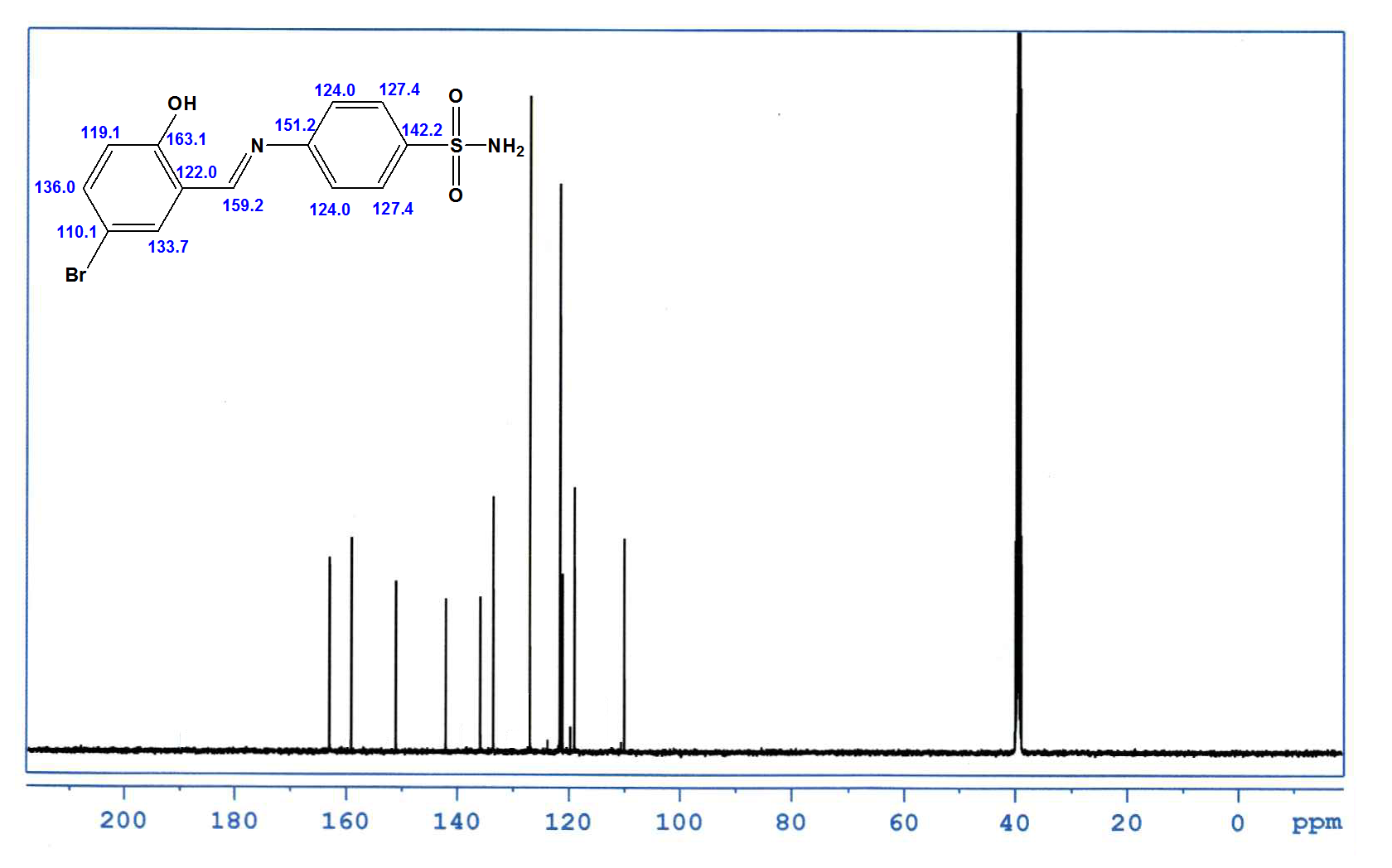
**

**Figure S8.** ^31^C NMR spectra of SB^1^ in DMSO-*d*_6_

**
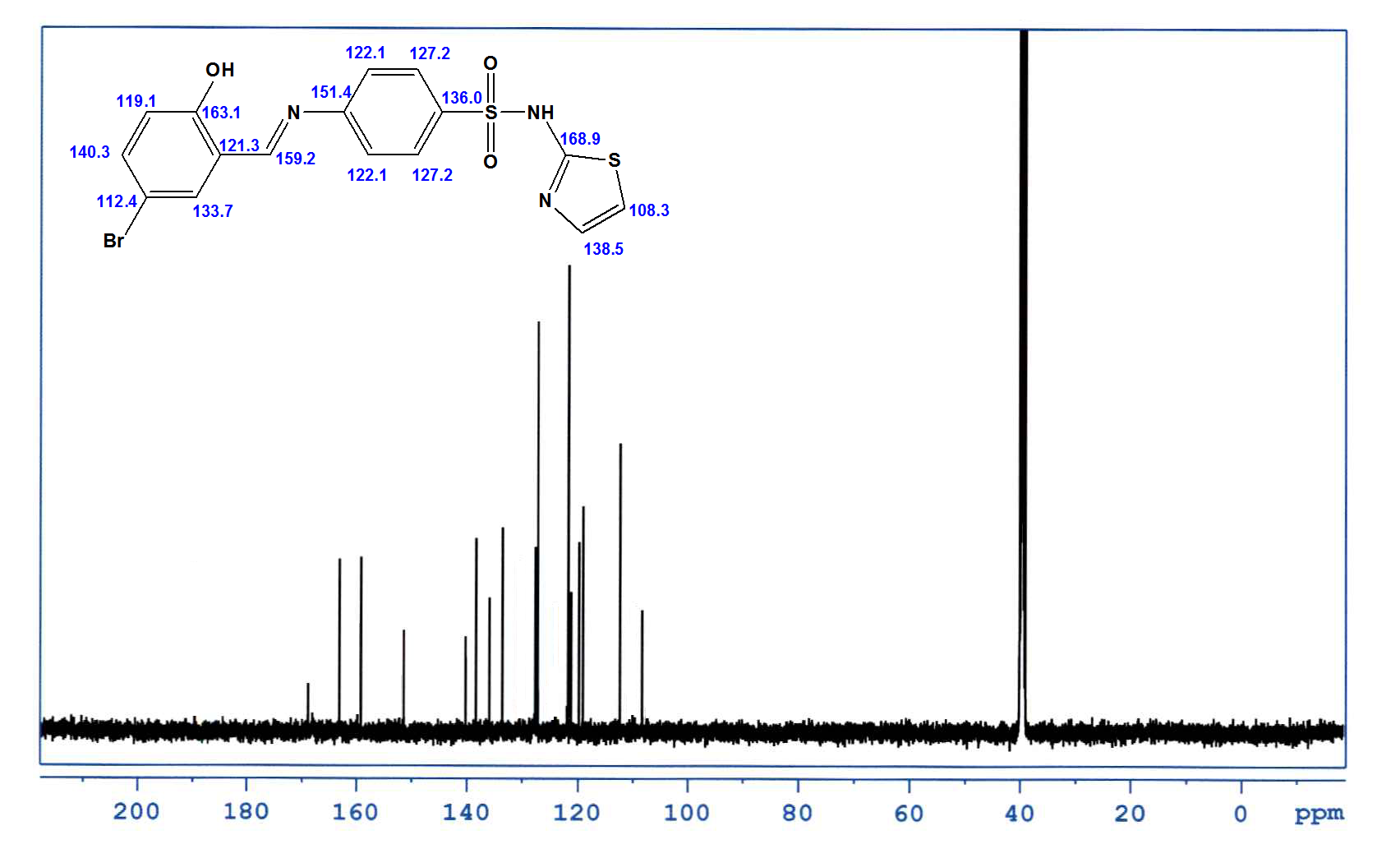
**

**Figure S9.** ^31^C NMR spectra of SB^2^ in DMSO-*d*_6_

**
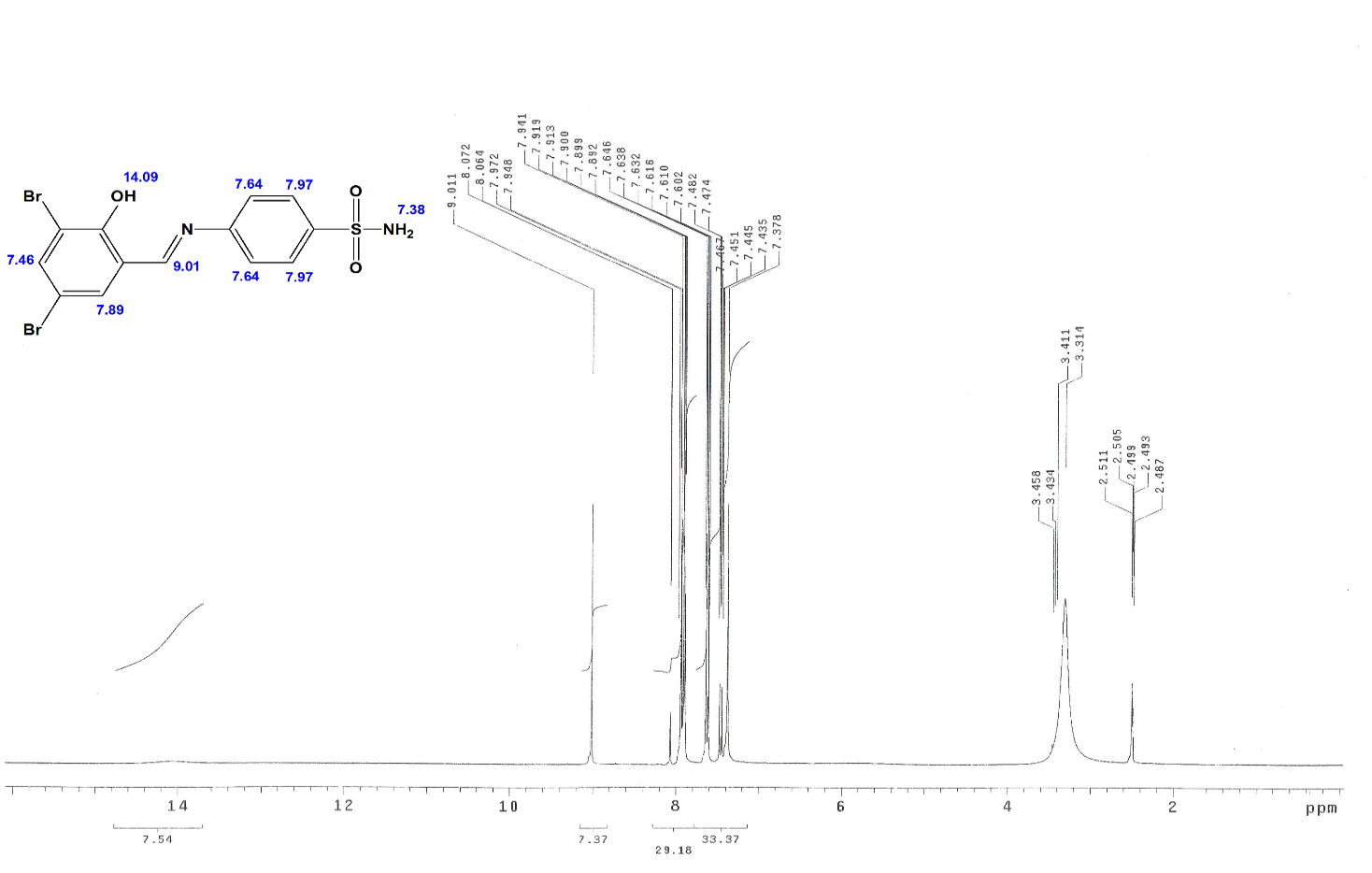
**

**Figure S10.** ^1^H NMR spectra of SB^3^ in DMSO-*d*_6_

**
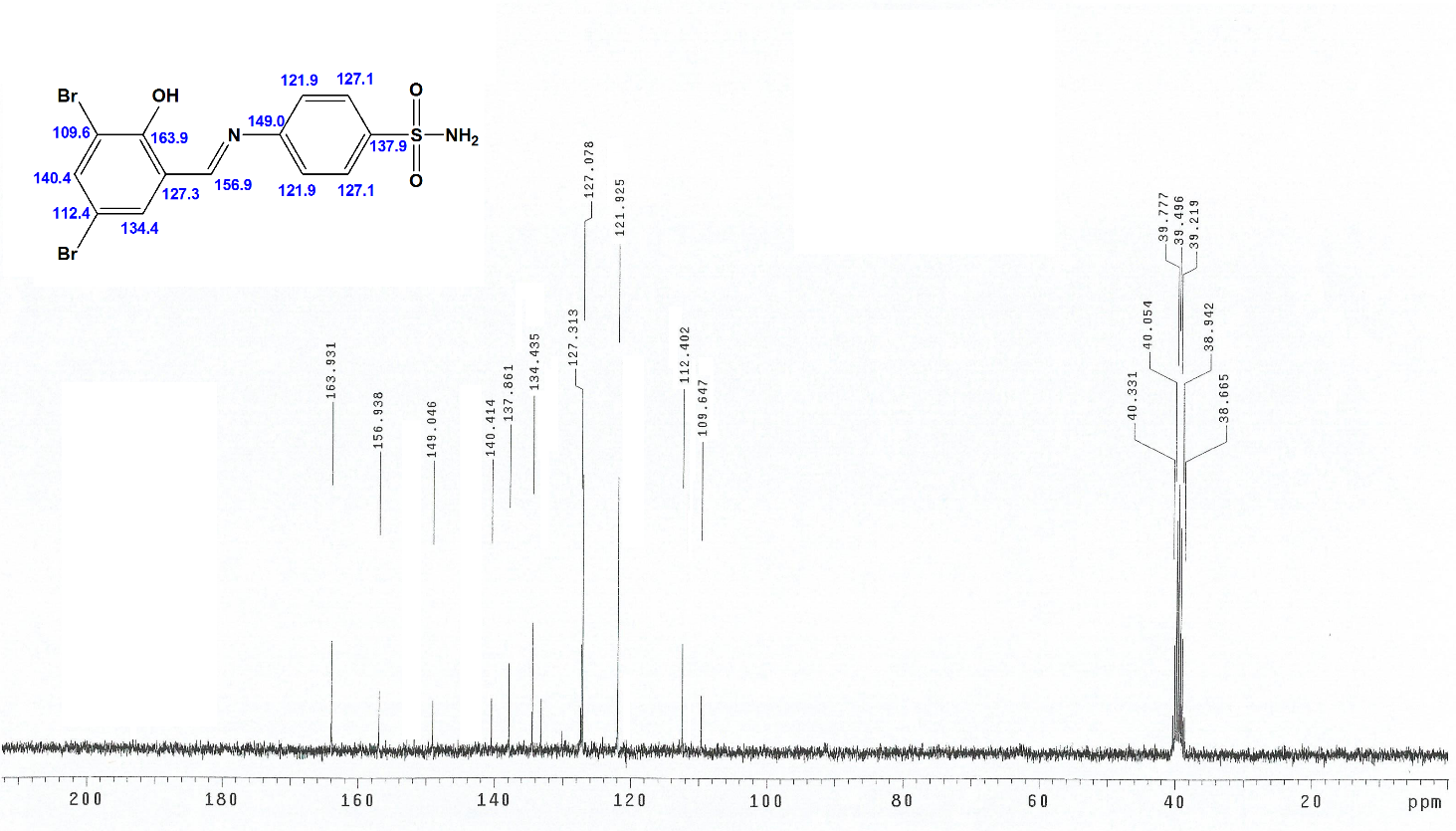
**

**Figure S11.** ^31^C NMR spectra of SB^3^ in DMSO-*d*_6_

**
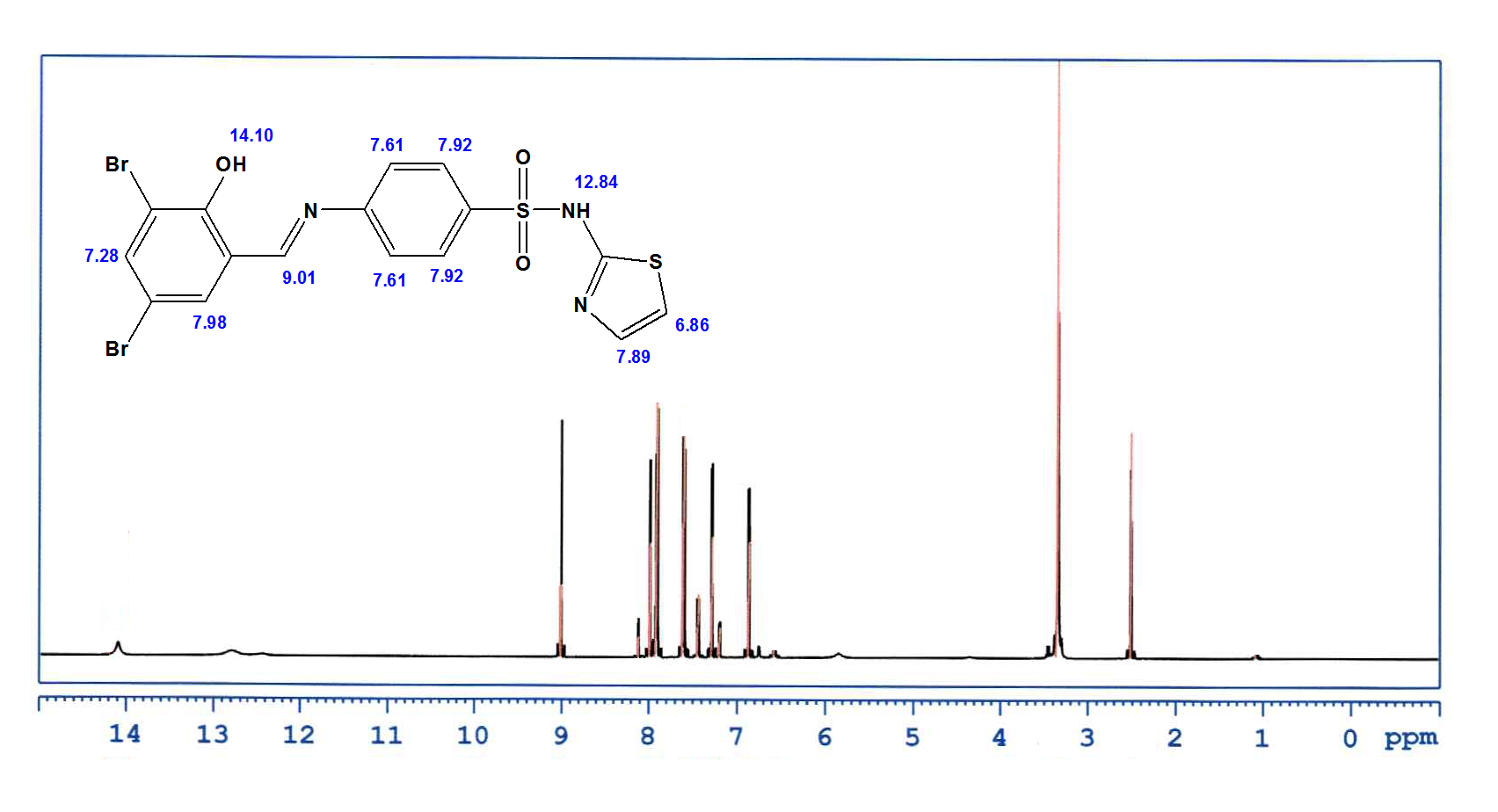
**

**Figure S12.** ^1^H NMR spectra of SB^4^ in DMSO-*d*_6_

**
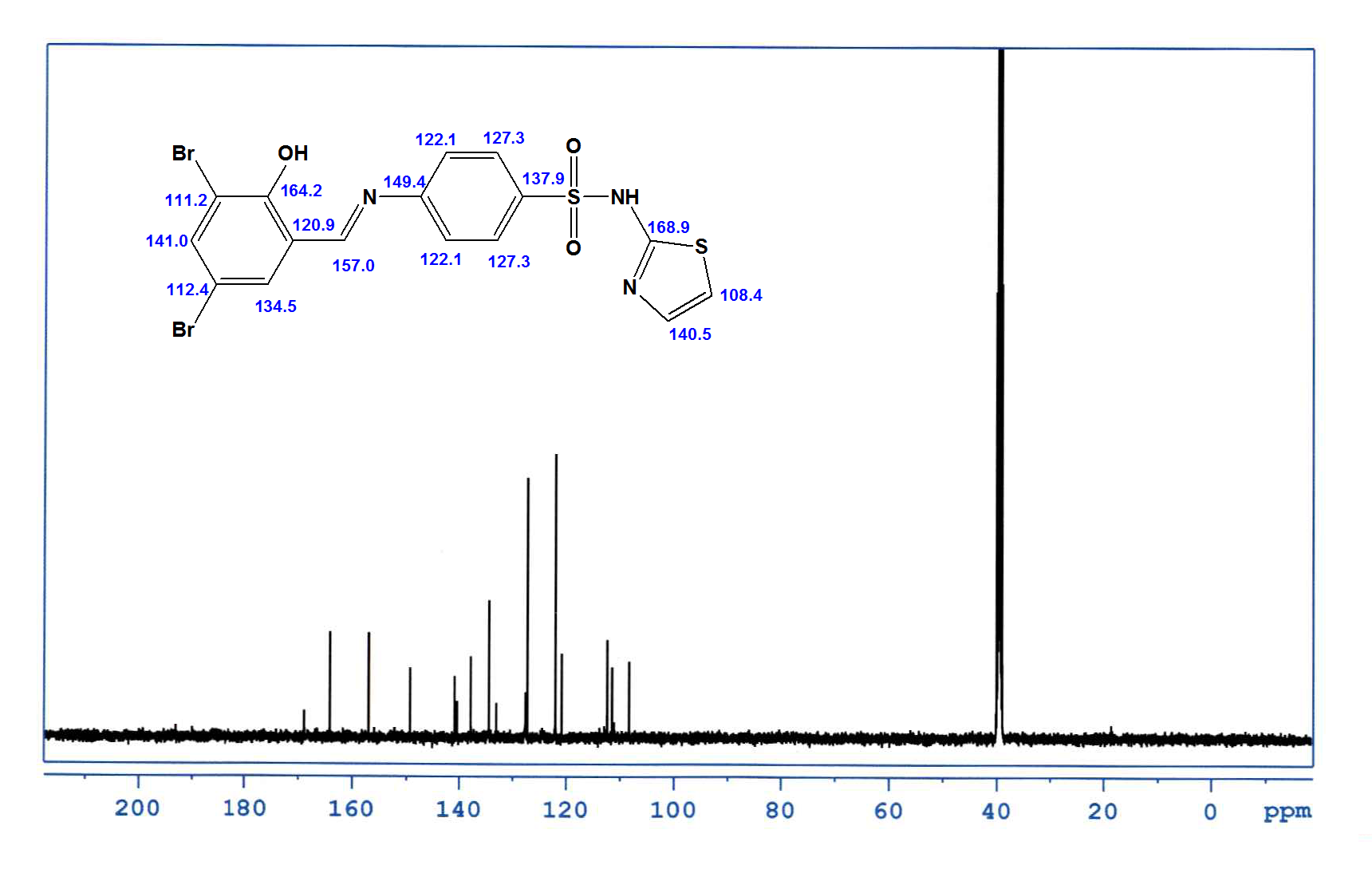
**

**Figure S13.** ^31^C NMR spectra of SB^4^ in DMSO-*d*_6_

**
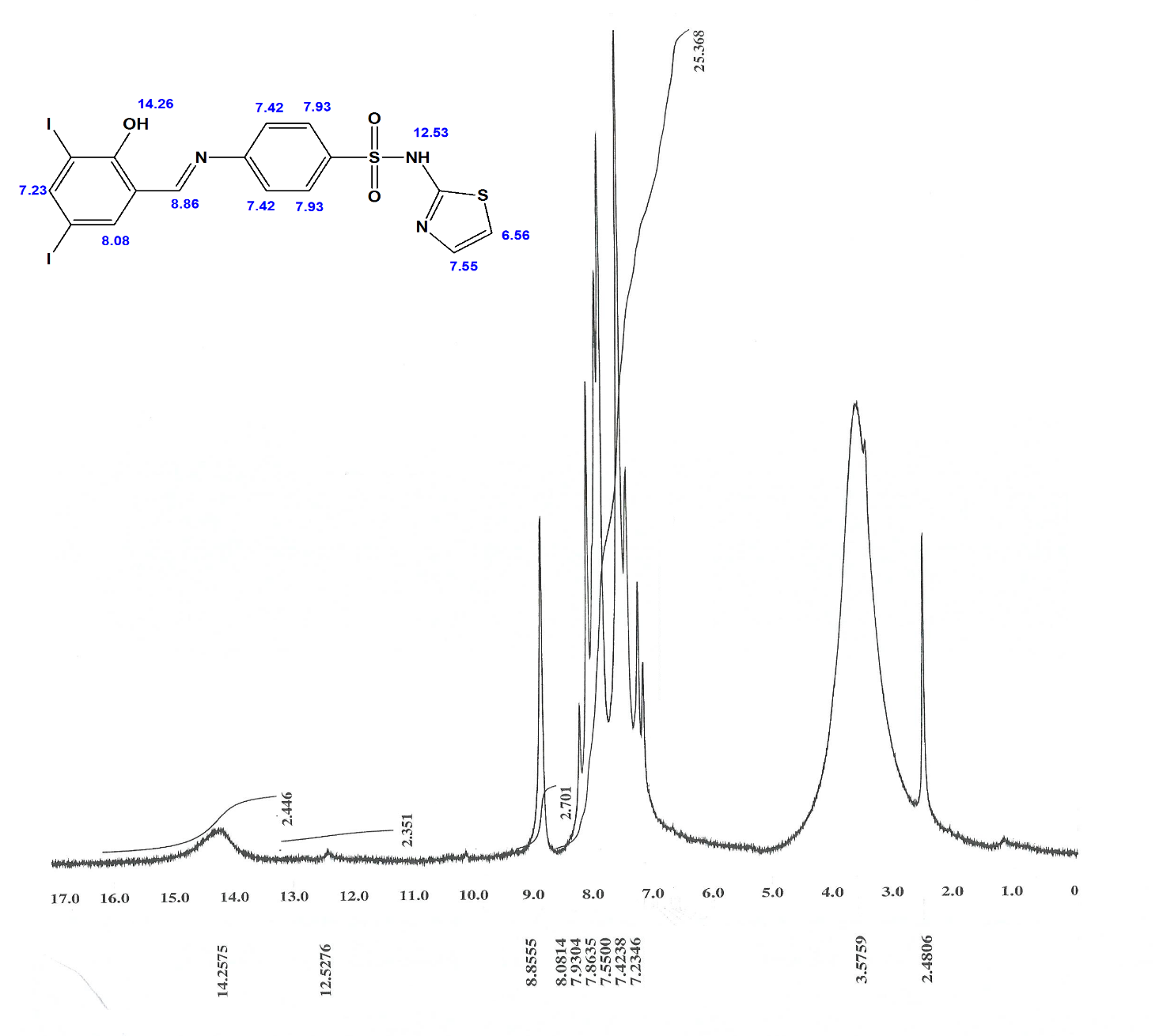
**

**Figure S14.** ^1^H NMR spectra of SB^5^ in DMSO-*d*_6_

**
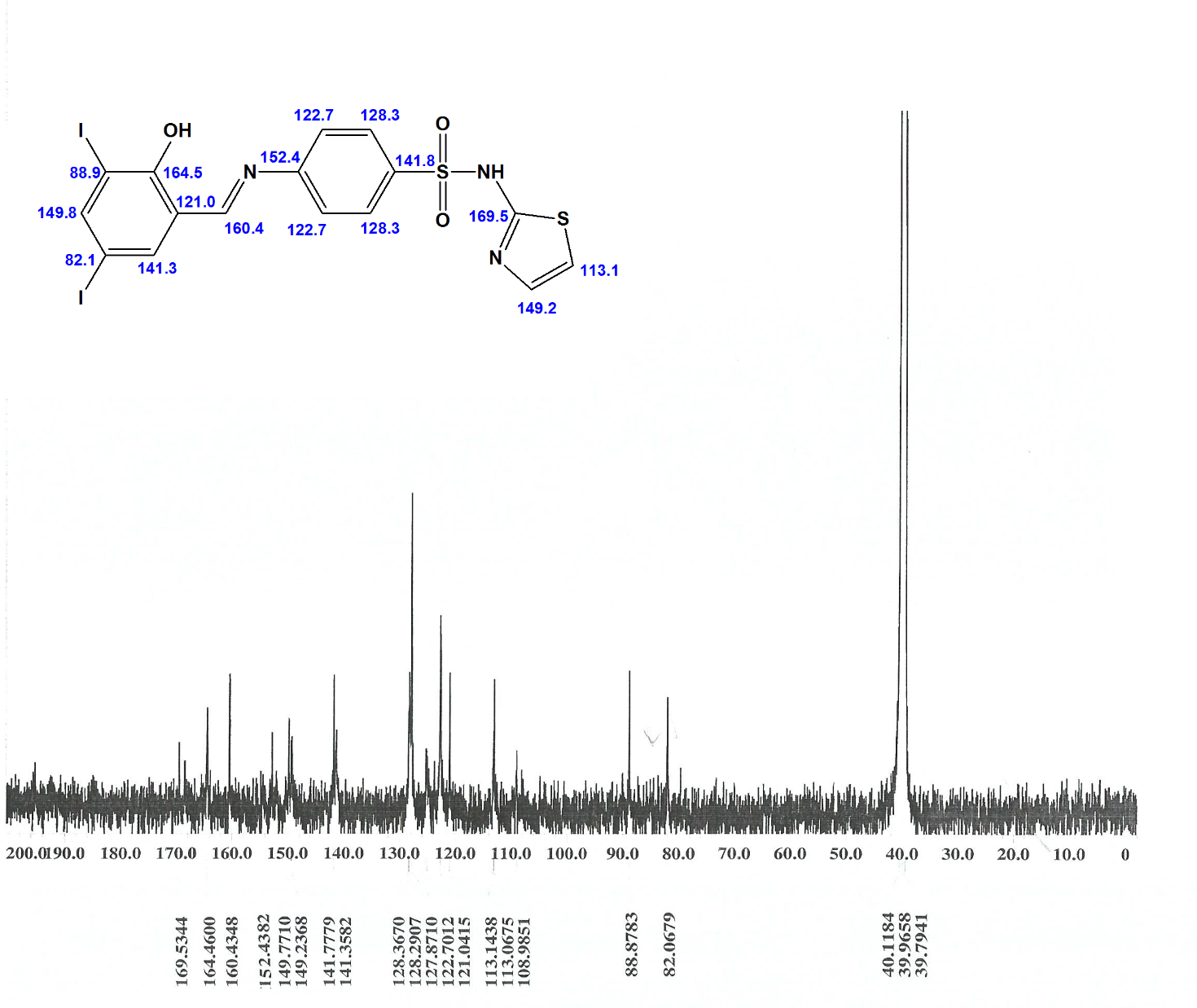
**

**Figure S15.** ^31^C NMR spectra of SB^5^ in DMSO-*d*_6_


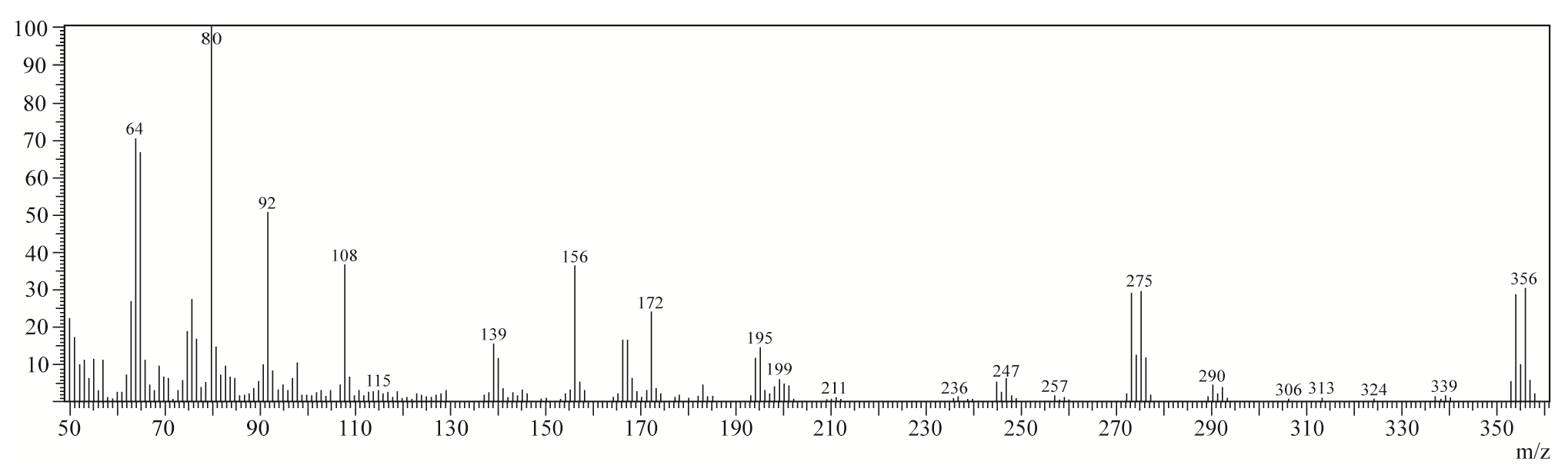


**Figure S16.** Mass spectrum of SB^1^


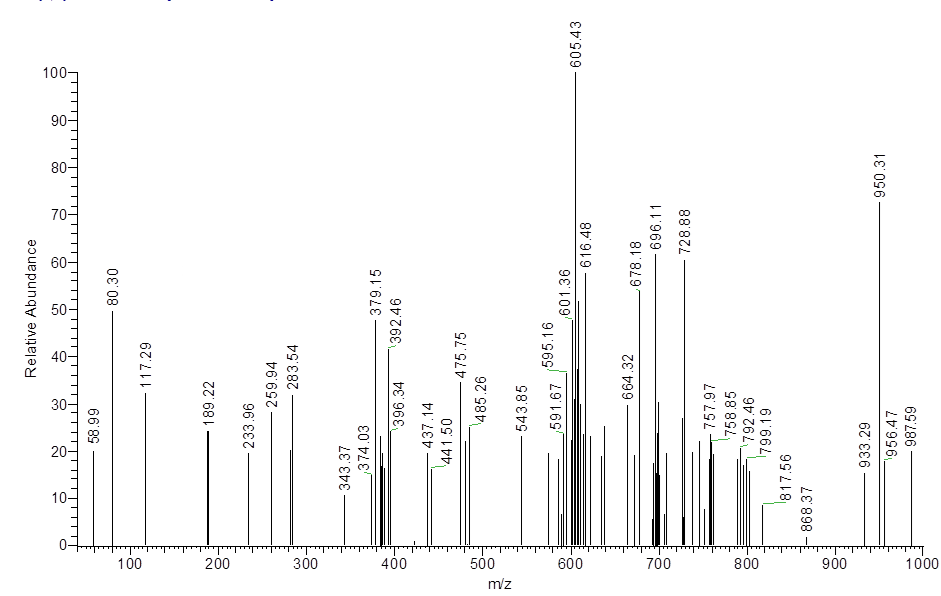


**Figure S17.** Mass spectrum of [Ni(SB^2^-H)_2_)].3H_2_O complex

**
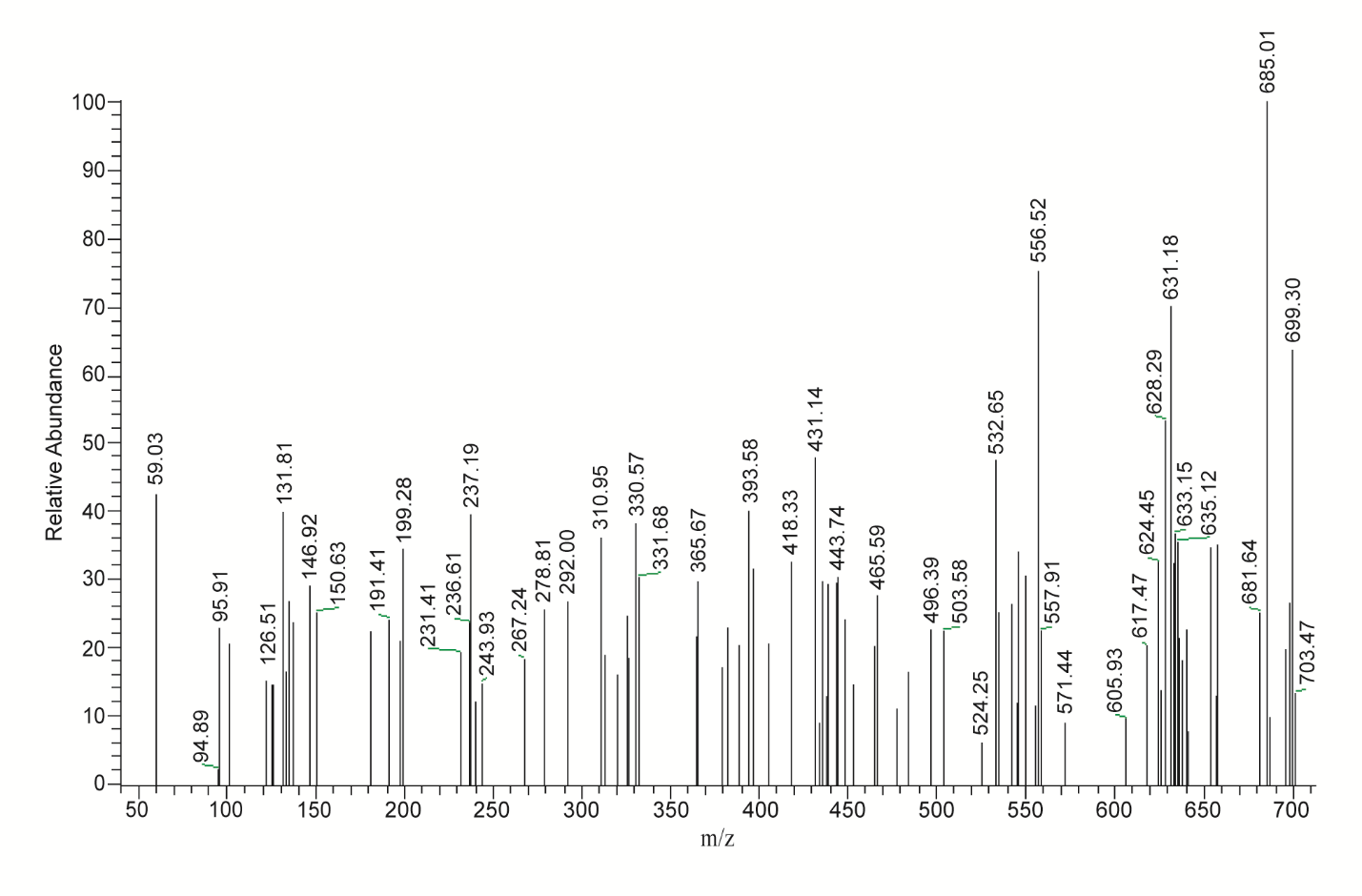
**

**Figure S18.** Mass spectrum of [Ni(SB^5^-H)(OH)(H_2_O)] complex


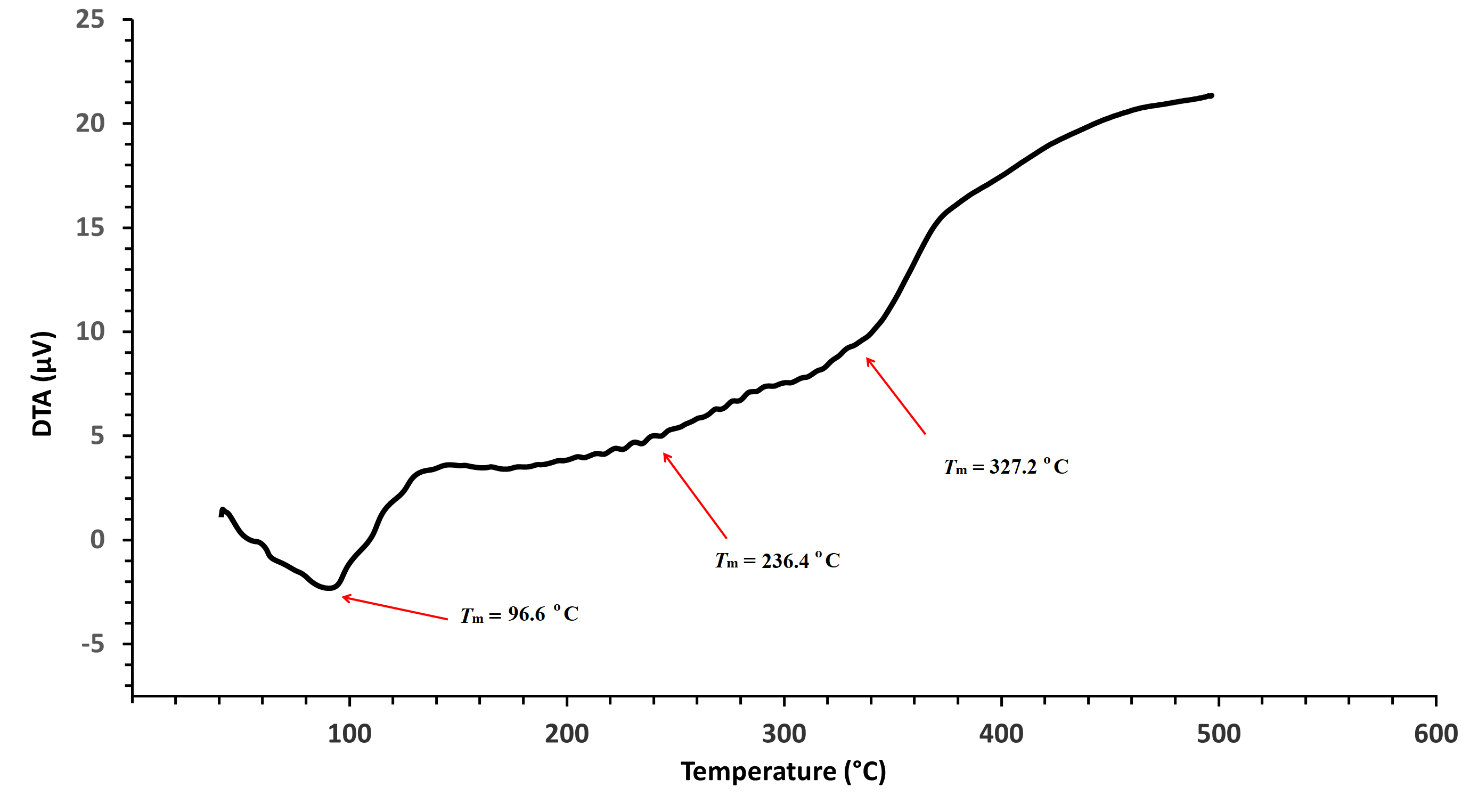


**Figure S19.** DTA of SB^5^ under N_2_ atmosphere


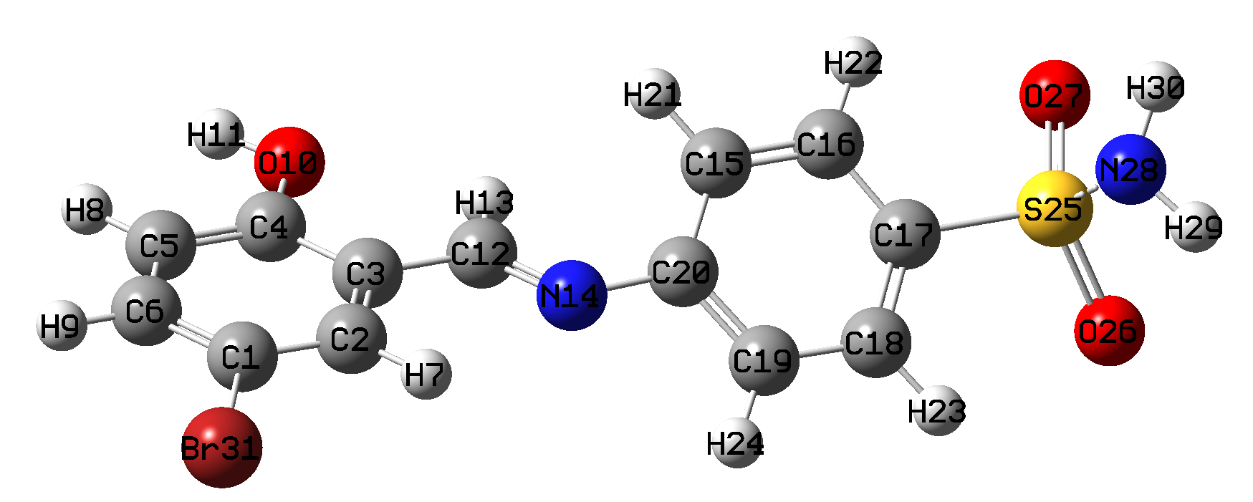


**Figure S20.** Optimized structures of SB^1^ using DFT-B3LYP/ Lanl2dz method, by GAUSSIAN 09 software version 9.5 and GAUSSVIEW 6.0.16.


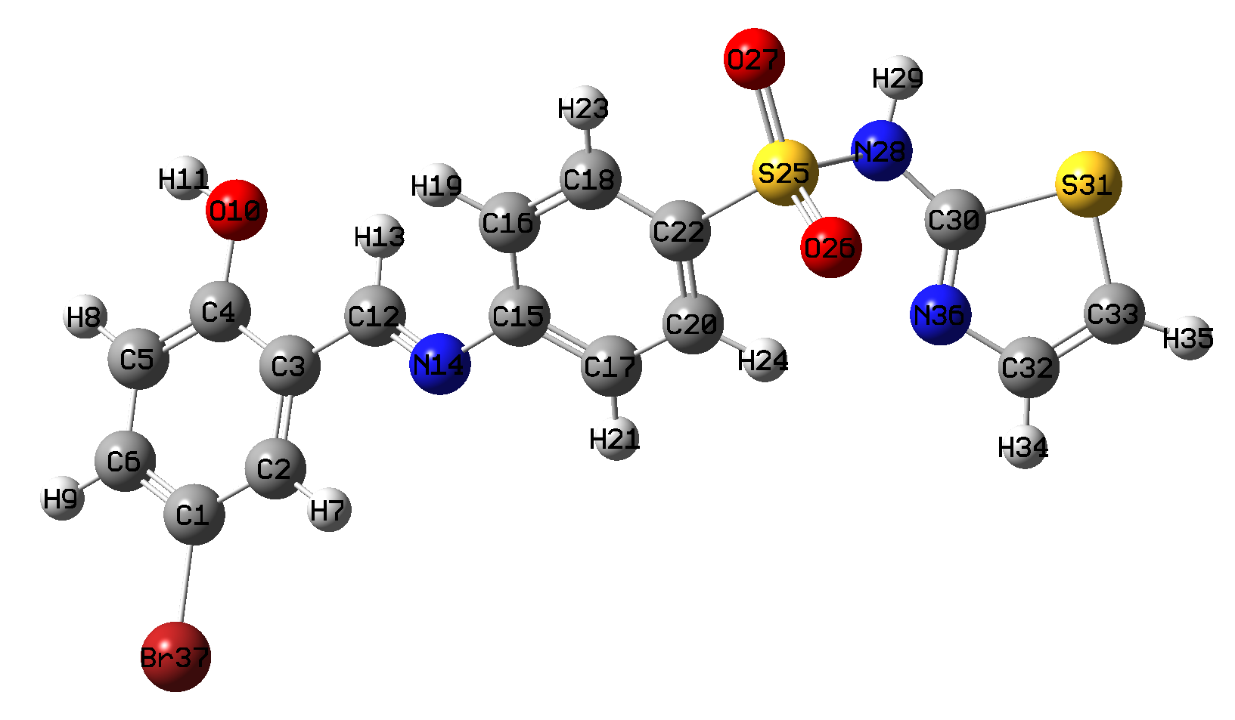


**Figure S21.** Optimized structures of SB^2^ using DFT-B3LYP/Lanl2dz method, by GAUSSIAN 09 software version 9.5 and GAUSSVIEW 6.0.16.


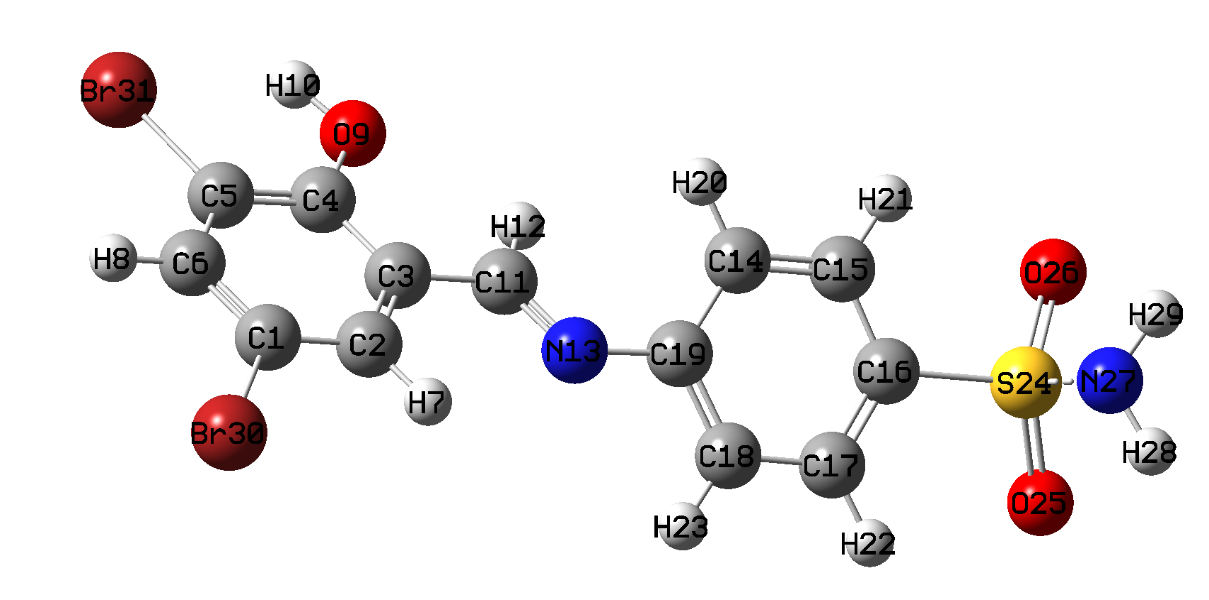


**Figure S22.** Optimized structure of SB^3^ using DFT-B3LYP/Lanl2dz method, by GAUSSIAN 09 software version 9.5 and GAUSSVIEW 6.0.16.


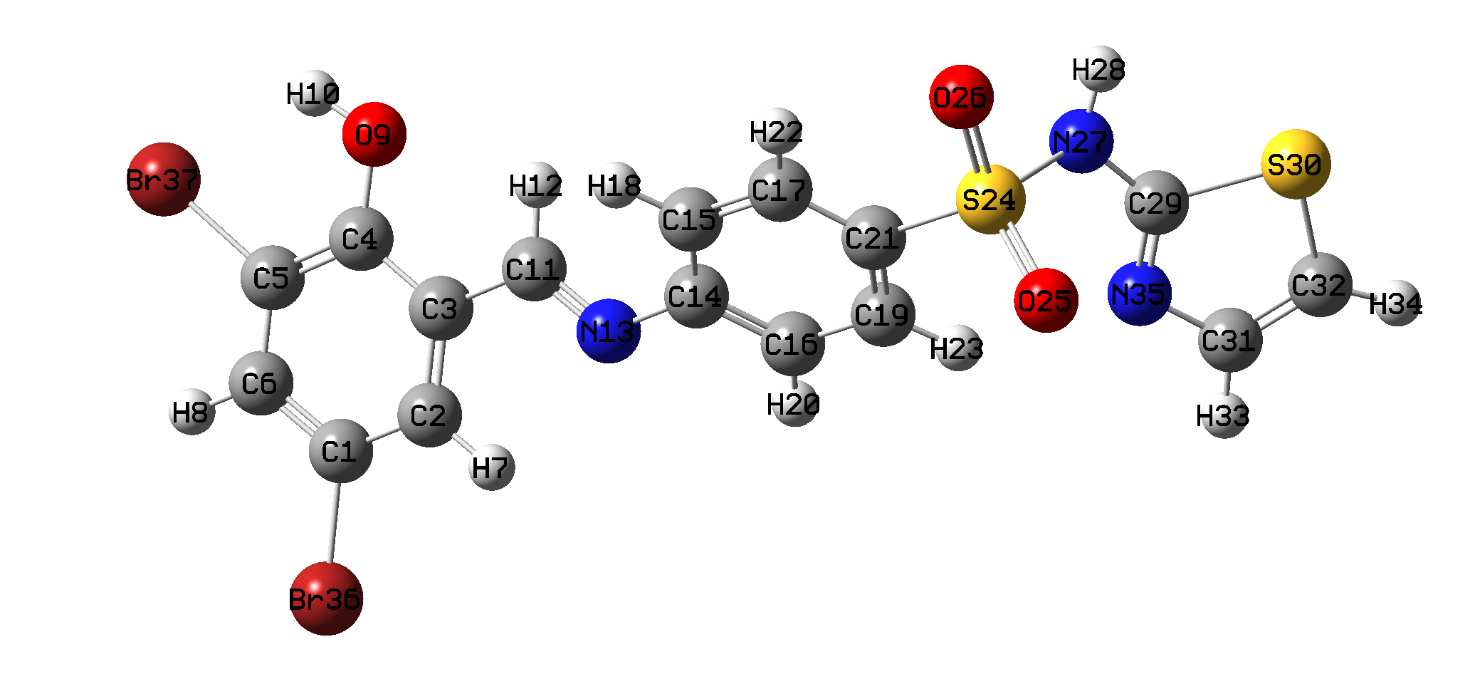


**Figure S23.** Optimized structure of SB^4^ using DFT-B3LYP/Lanl2dz method, by GAUSSIAN 09 software version 9.5 and GAUSSVIEW 6.0.16.


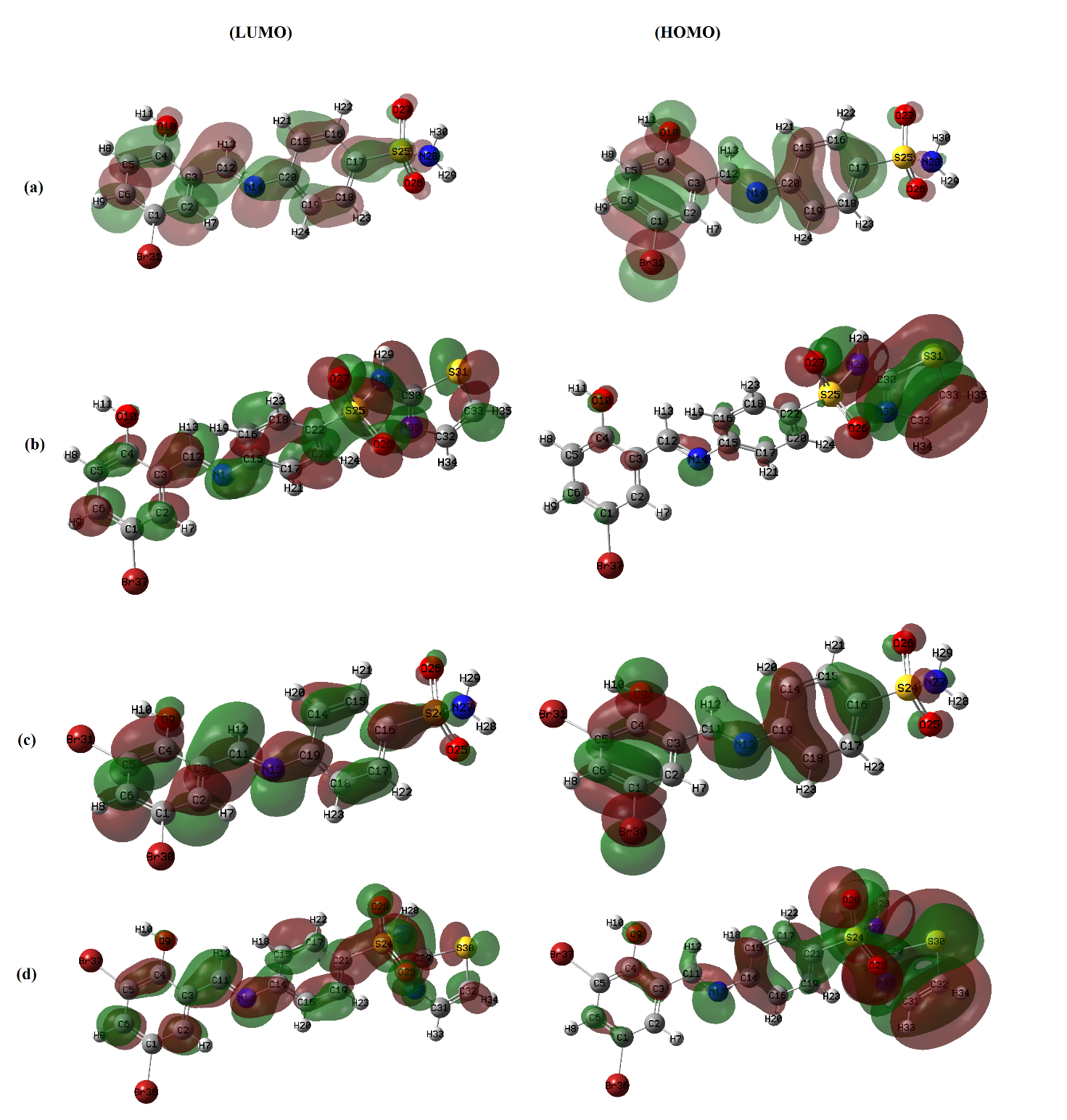


**Figure S24.** LUMO and HOMO of (a) SB^1^, (b) SB^2^, (c) SB^3^, and (d) SB^4^, using GAUSSIAN 09 software version 9.5 and GAUSSVIEW 6.0.16.

**
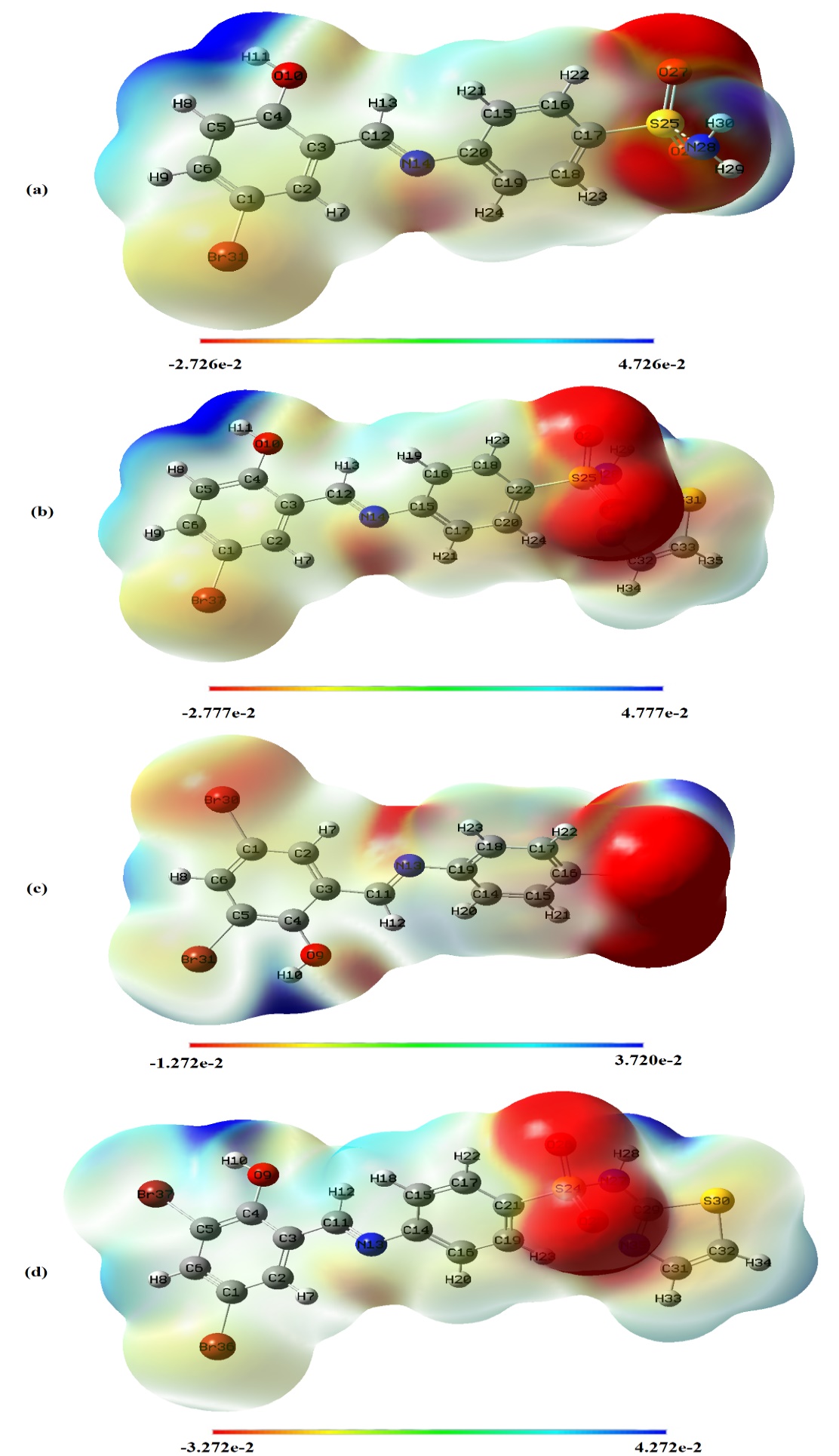
**

**Figure S25.** Molecular electrostatic potential map of (a) SB^1^, (b) SB^2^, (c) SB^3^, and (d) SB^4^, using GAUSSIAN 09 software version 9.5 and GAUSSVIEW 6.0.16.


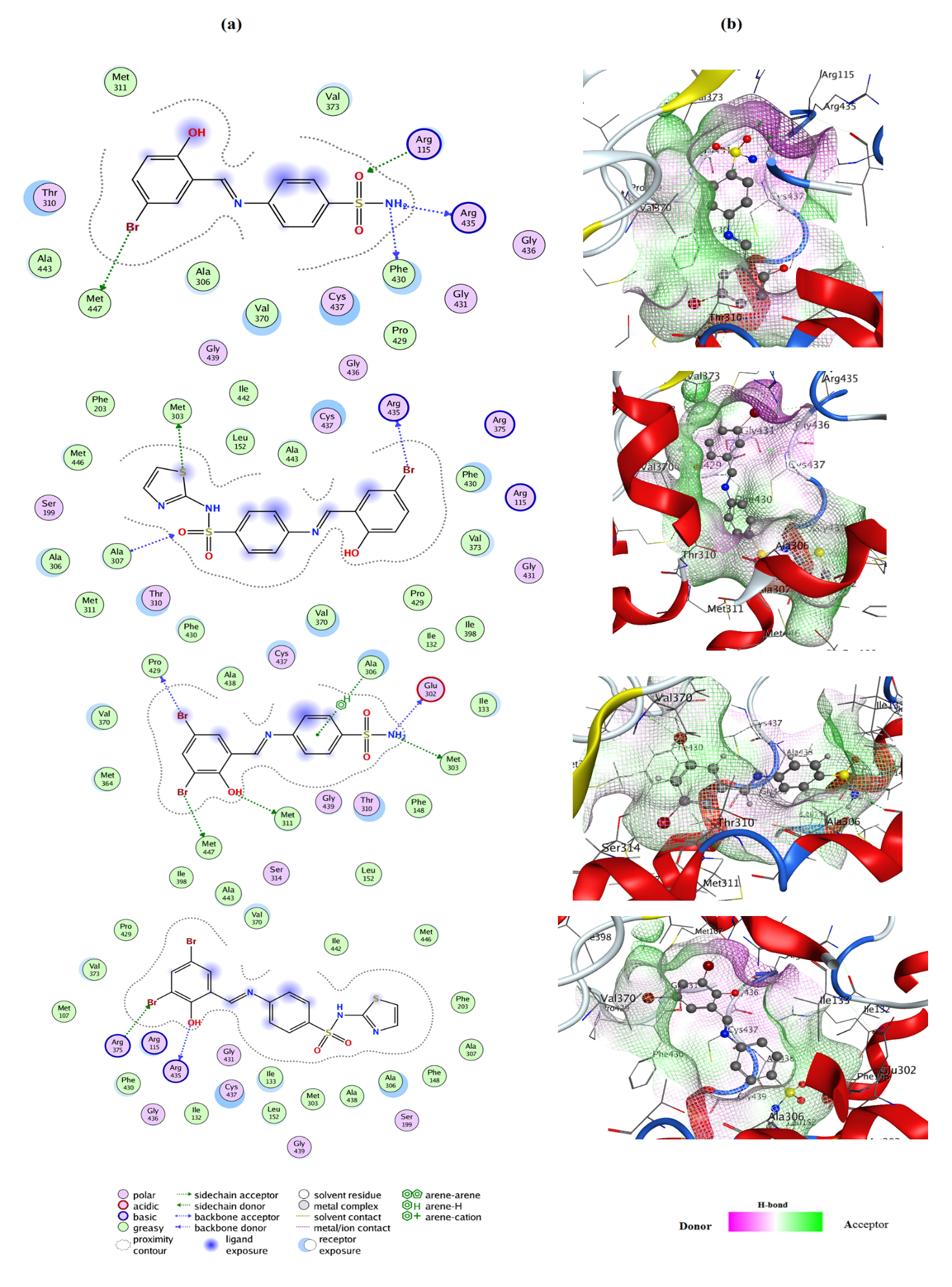


**Figure S26.** Binding features (a) surface maps (b) best docked poses of the synthesized ligands (SB^1^, SB^2^, SB^3^, and SB^4^) against breast cancer protein *3s7s*, using the (MOE 2015.10) software.


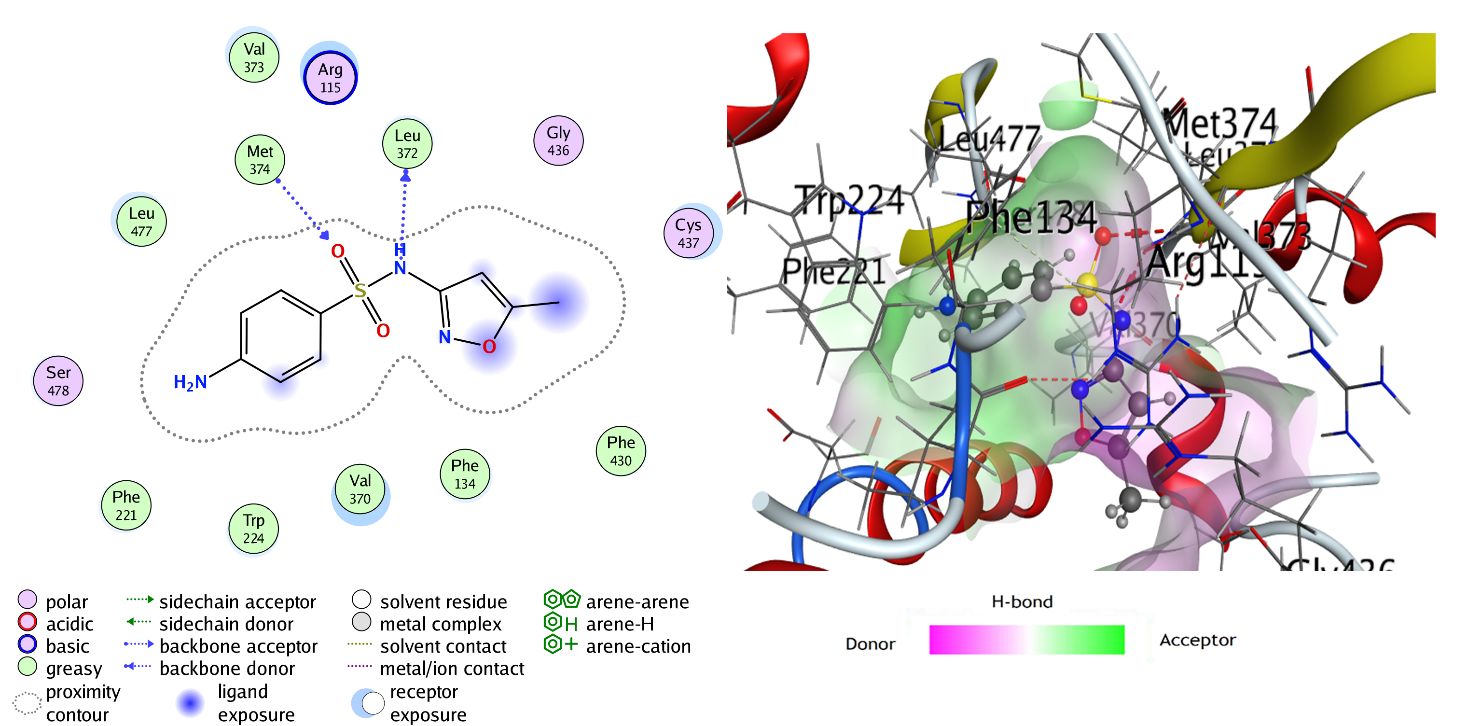


**Figure S27.** Binding features (a) surface maps (b) best-docked poses of the standard drug Trimethoprim-sulfamethoxazole (Bactrim) against breast cancer protein *3s7s*, using the (MOE 2015.10) software.

**Figure S28.** The inhibitory dose-response curves of selected Ni(II) complexes against MCF-7

**Figure S29.** The inhibitory dose-response curves of SB^4^, Ni(II)-SB^2^ and Ni(II)-SB^4^ and cisplatin against normal human oral epithelial OEC.

| **Compound** | **ν(NH_2_)** | **ν(NH)/ ν(OH)** | **ν(C=C)** | ***v*(C=N)** | **ν_asym_(SO_2_)** | ***v*(C-O)** | ***v*_sym_(SO_2_)** | **ν(M-O)** | **ν(M-N)** |
| --- | --- | --- | --- | --- | --- | --- | --- | --- | --- |
| **SB^1^** | 3359  3270 | 3140 | 1616 | 1559 | 1336 | 1273 | 1154 | --- | --- |
| **[Ni(SB^1^)_2_(SB^1^-H)].2H_2_O** | 3356  3264 | 3493b  3155w | 1625 | 1558 | 1331 | 1236sh | 1154 | 537 | 485 |
| **SB^2^** | --- | 3138  3089 | 1619 | 1565  1525 | 1318 | 1274 | 1148 | --- | --- |
| **[Ni(SB^2^)_2_].3H_2_O** | --- | 3303  3129  3086 | 1617 | 1552 | 1314 | 1231w | 1141 | 506 | 458 |
| **SB^3^** | 3314  3232 | 3053 | 1623 | 1584 | 1322 | 1232 | 1157 | --- | --- |
| **[Ni(SB^3^-H)(OH)(H_2_O)]** | 3326  3289sh | 3442b  3064  3441 | 1611 | 1577sh | 1321 | 1209 | 1155 | 512 | 490 |
| **SB^4^** | --- | 3138  3092 | 1613 | 1574  1542 | 1331 | 1279 | 1142 | --- | --- |
| **[Ni(SB^4^-H)_2_].4H_2_O** | --- | 3579  3363b  3147  3097 | 1610 | 1581  1530 | 1319 | 1246w | 1141 | 566 | 506 |
| **SB^5^** | --- | 3457  3356  3099 | 1613 | 1579  1533 | 1327 | 1276 | 1138 | --- | --- |
| **[Ni(SB^5^-H)(OH)(H_2_O)]** | --- | 3441b  3367  3315  3087sh | 1607 | 1571sh  1532w | 1320 | 1236w | 1138 | 516 | 488 |

b = broad, sh = shoulder, w = weak.

**Table S1** IR spectra (4000-400 cm^-1^) of ligands (SB^1^-SB^5^) and their Ni(II) complexes

| **Angle** | **Bond** | **length (Å)** | **Bond** | **Complex** |
| --- | --- | --- | --- | --- |
| 95.38 | N(12)–Ni(34)–O(9) | 1.77 | Ni(34)–N(12) | **[Ni(SB^5^-H)(OH)(H_2_O)]** |
| 87.47 | O(9)–Ni(34)–O(36) | 1.76 | Ni(34)–O(9) |  |
| 78.36 | O(36)–Ni(34)–O(35) | 1.79 | Ni(34)–O(35) |  |
| 98.97 | O(35)–Ni(34)–N(12) | 1.86 | Ni(34)–O(36) |  |
|  |  | 1.44 | N(12)–C(13) |  |
|  |  | 1.33 | N(12)=C(10) |  |
|  |  | 1.37 | O(9)–C(4) |  |
|  |  | 1.43 | C(4)=C(3) |  |
|  |  | 1.58 | S(23)=O(24) |  |
|  |  | 1.80 | S(23)–N(26) |  |
|  |  | 1.29 | N(33)=C(27) |  |
|  |  | 1.86 | S(28)–C(27) |  |
|  |  | 1.41 | N(13)–C(14) | **SB^5^** |
|  |  | 1.29 | N(13)=C(11) |  |
|  |  | 1.31 | O(9)–C(4) |  |
|  |  | 1.41 | C(4)–C(3) |  |
|  |  | 1.40 | C(4)=C(5) |  |
|  |  | 1.58 | S(24)=O(25) |  |
|  |  | 1.59 | S(24)=O(26) |  |
|  |  | 1.84 | S(24)–N(27) |  |
|  |  | 1.30 | N(35)=C(29) |  |
|  |  | 1.87 | S(30)–C(29) |  |

**Table S2** Selected bond lengths (Å) and bond angles of [Ni(SB^5^-H)(OH)(H_2_O)] complex and SB^5^
